# Breastfeeding and impact on childhood hospital admissions: a nationwide birth cohort in South Korea

**DOI:** 10.1038/s41467-023-41516-y

**Published:** 2023-09-20

**Authors:** Jeong-Seon Lee, Jae Il Shin, Sunyeup Kim, Yong-Sung Choi, Youn Ho Shin, Jimin Hwang, Jung U Shin, Ai Koyanagi, Louis Jacob, Lee Smith, Han Eol Jeong, Yunha Noh, In-Sun Oh, Sang Youl Rhee, Chanyang Min, Seong Ho Cho, Steve Turner, Guillaume Fond, Laurent Boyer, Dong In Suh, Krishna Prasad Acharya, Ju-Young Shin, Seung Won Lee, Dong Keon Yon

**Affiliations:** 1 Department of Pediatrics, Soonchunhyang University Bucheon Hospital, Soonchunhyang University School of Medicine, Bucheon, Republic of Korea; 2 Department of Pediatrics, Yonsei University College of Medicine, Seoul, Republic of Korea; 3 Department of Precision Medicine, Sungkyunkwan University School of Medicine, Suwon, Republic of Korea; 4 Department of Pediatrics, Kyung Hee University Medical Center, Kyung Hee University College of Medicine, Seoul, Republic of Korea; 5 Department of Pediatrics, The Catholic University of Korea, Yeouido St. Mary’s Hospital, Seoul, Republic of Korea; 6 Department of Epidemiology, Johns Hopkins Bloomberg School of Public Health, Baltimore, MD USA; 7 Department of Dermatology, CHA Bundang Medical Center, CHA University School of Medicine, Seongnam, Republic of Korea; 8 Research and Development Unit, Parc Sanitari Sant Joan de Déu, CIBERSAM, ISCIII, Barcelona, Spain; 9 Department of Physical Medicine and Rehabilitation, Lariboisière-Fernand Widal Hospital, AP-HP, Université Paris Cité, Paris, France; 10 Centre for Health, Performance and Wellbeing, Anglia Ruskin University, Cambridge, UK; 11 School of Pharmacy, Sungkyunkwan University, Suwon, Republic of Korea; 12 Deparments of Epidemiology, Biostatistics, and Occupational Health, McGill University, Montreal, Canada; 13 Centre for Clinical Epidemiology, Lady Davis Institute, Jewish General Hospital, Montreal, Canada; 14 Center for Digital Health, Medical Science Research Institute, Kyung Hee University Medical Center, Kyung Hee University College of Medicine, Seoul, Republic of Korea; 15 Division of Allergy-Immunology, University of South Florida Morsani College of Medicine, Tampa, FL USA; 16 Maternity and Child Health Division, NHS Grampian, Aberdeen, UK; 17 CEReSS-Health Service Research and Quality of Life Center, Assistance Publique-Hopitaux de Marseille, Aix-Marseille University, Marseille, France; 18 Department of Pediatrics, Seoul National University Hospital, Seoul National University College of Medicine, Seoul, Republic of Korea; 19 Animal Quarantine Office Kathmandu, Budhanilkantha, Kathmandu Nepal; 20 Department of Biohealth Regulatory Science, Sungkyunkwan University, Suwon, Republic of Korea

**Keywords:** Paediatric research, Epidemiology, Public health

## Abstract

Benefits of breastfeeding for both the mother and the child are well established, but a comprehensive and robust study to investigate the protective effect of breastfeeding and attenuated time effect stratified by cause of morbidity are lacking. This study is based on the nationwide birth cohort in Korea that includes data on all infants born from 2009 to 2015. Of 1,608,540 children, the median follow-up period was 8.41 years (interquartile range, 6.76-10.06). When compared to children with fully formula feeding, the hospital admission rate was 12% lower in those with partially breastfeeding and 15% lower in those with exclusive breastfeeding. The apparent protective effect of breastfeeding was reduced with increasing age. Our study provides potential evidence of the beneficial association of breastfeeding on subsequent hospital admissions. The protective effect declined over time as the children grew older. Encouraging any breastfeeding for at least the first 6 months among infants is an important public health strategy to improve overall child health.

## Abstract (web summary)

Benefits of breastfeeding are well established, but a comprehensive study about its impacts on hospitalizations is lacking. Here, the authors use Korean nationwide birth cohort data (n = 1,608,540) and find that breastfeeding for at least 6 months was associated with a lower risk for subsequent hospital admissions.

## Introduction

The World Health Organization (WHO) and United Nations International Children’s Emergency Fund (UNICEF) recommend that children initiate breastfeeding within the first hour of birth and be exclusively breastfed for the first 6 months of life (EBF-6)^1^. Although breastfeeding recommendations have evolved remarkably in low- and middle-income countries, there is less consensus on the importance of breastfeeding promotion in high-income countries^2^. Breastfeeding has been found to be beneficial for mothers by aiding in the prevention of breast cancer, diabetes, and ovarian cancer, as well as lowering infectious disease morbidity and aiding in better academic performance in the offspring^2^. There were some studies on the relationship between breastfeeding and antibody responses to vaccine^3,4^. In those studies, antibody levels were significantly higher in the breast-fed vs non-breast-fed infants. Breastfeeding modulates the microbiota, which may induce regulatory T cells involved in in T helper (Th)1/Th2 balance and enhance systemic innate immunity^5,6^. In addition increased omega-3 essential polyunsaturated fatty acid and soluble CD14 concentrations in breast milk have been shown to associate with a lower risk of eczema development^7,8^. Those studies explain the protective effects of breastfeeding in infant’s immunity. However, the effects of breastfeeding on the hospitalization rates remain unknown. Many previous studies have investigated the protective effect of breastfeeding on the subsequent hospital admission^9–16^. However, there is a lack of a comprehensive approach and application of a robust study design to investigate the protective effect of breastfeeding and attenuated time effect stratified by cause of morbidity.

In this work, we established the hypothesis that breastfeeding may reduce the risk of subsequent hospital admission rate. Therefore, we aimed to investigate the potential association between breastfeeding and risk of subsequent hospital admission rate and attenuation of protective effect over time through a population-based nationwide birth cohort in Korea.

## Results

### Descriptive overview

**Fig. 1:**
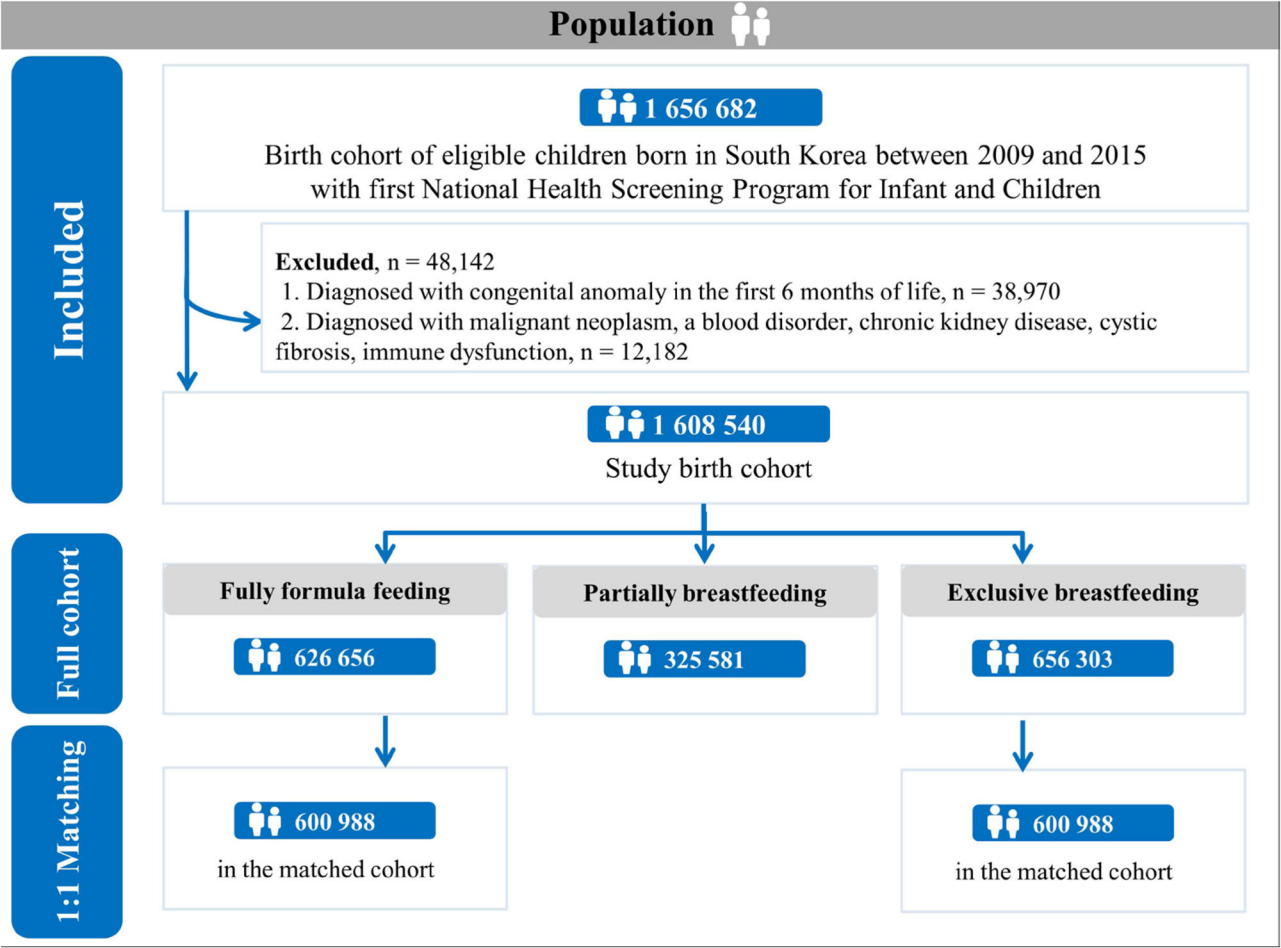
Diagrammatic representation of Korean nationwide birth cohort before and after propensity matching. Total 1,608,540 participants born in South Korea between 2009 and 2015 were included. According to the types of infant feeding, they were categorized in three groups: exclusive breastfeeding, partially breastfeeding, and fully formula feeding. Propensity scores were derived using a multivariable logistic regression model from the predicted probability of exclusively breastfed infants vs. fully formula-fed infants (each *n* = 600,988).

**Fig. 2:**
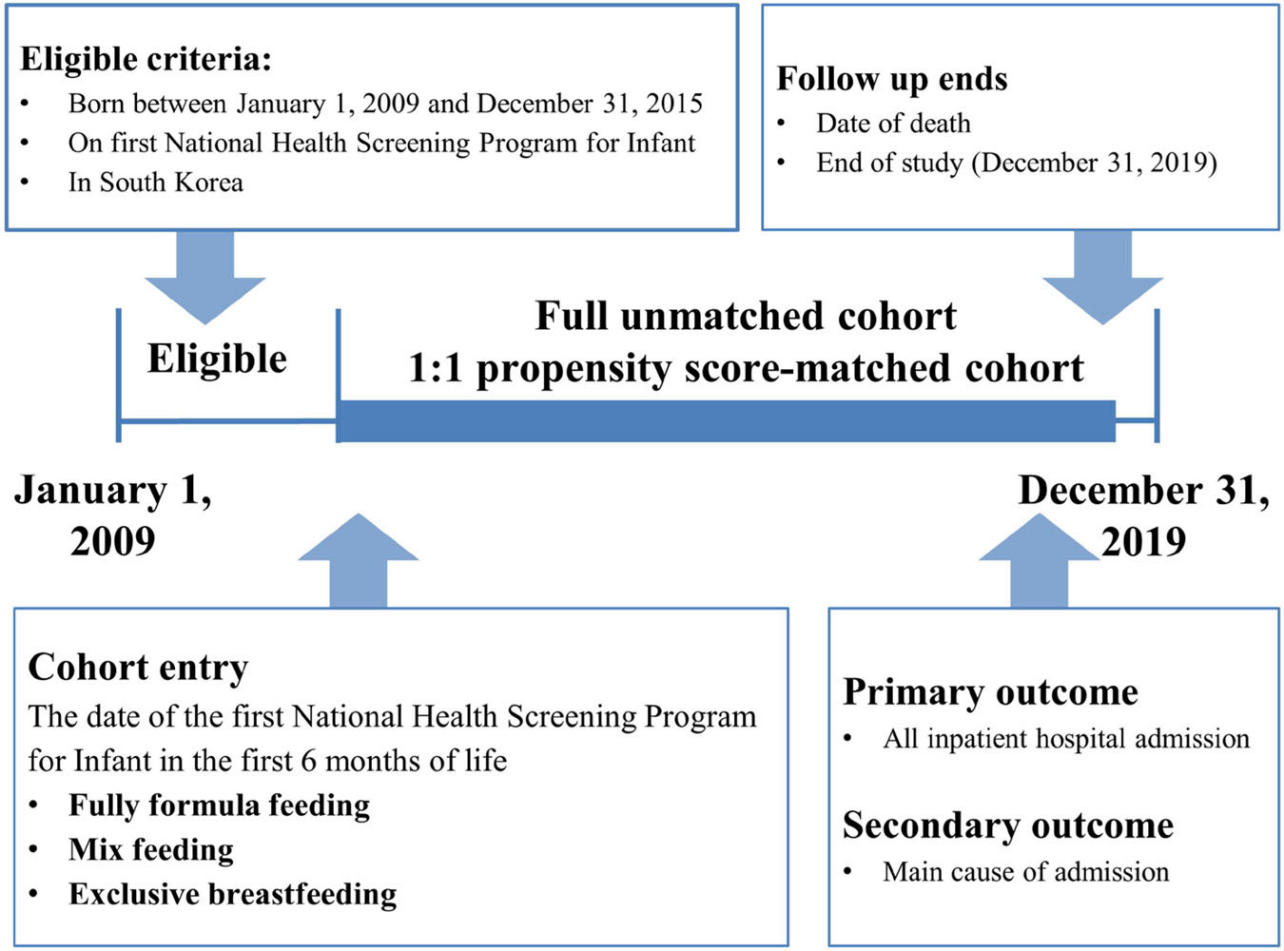
Flow chart of study enrollment. Among 2,010,325 infants, 401,785 infants were excluded and 1,608,540 infants were included. The observation period was between 1 January 2009 and 31 December 2019.

The sociodemographic characteristics of 1,608,540 participants are described in Table 1, Supplementary Table 1, and Figs. 1, 2. Of the infants, 49.7% were female, and 54.5% were rural residents. During the median follow-up period of 8.41 years (interquartile range, 6.76−10.06), 1,054,731 (65.6%) participants experienced any hospital admission during the follow-up period. We identified 626,656 (39.0%), 325,581 (20.2%), and 656,303 (40.8%) children with fully formula-feeding, partially breastfeeding, and exclusive breastfeeding, respectively. During the full follow-up period, the distribution of hospital admissions for infections, respiratory tract, gastrointestinal tract, oral cavity, mental health, injury/external causes, and genitourinary tract are described in Supplementary Table 2.

**Table 1:** Sociodemographic characteristics of participants in the Korean nationwide birth cohort (*n* = 1,608,540)

Characteristic	Full unmatched cohort (total)	Hospital admission	
		No admission	Any admission
Total, *n* (%)	1,608,540 (100.0)	553,809 (34.4)	1,054,731 (65.6)
Baseline characteristics			
Infant sex, *n* (%)			
Female	799,939 (49.7)	294,326 (53.2)	505,613 (47.9)
Male	808,601 (50.3)	259,483 (46.9)	549,118 (52.1)
Calendar period of birth, *n* (%)			
2009−2010	599,277 (37.3)	208,024 (37.6)	391,253 (37.1)
2011−2012	490,989 (30.5)	167,000 (30.2)	323,989 (30.7)
2013−2015	518,274 (32.2)	178,785 (32.3)	339,489 (32.2)
Birth season			
Spring (March−May)	401,371 (25.0)	138,985 (25.1)	262,386 (24.9)
Summer (June−August)	362,642 (22.5)	122,652 (22.1)	239,990 (22.8)
Autumn (September−November)	416,415 (25.9)	142,992 (25.8)	273,423 (25.9)
Winter (December−February)	428,112 (26.6)	149,180 (26.9)	278,932 (26.4)
Region of residence, *n* (%)			
Rural	877,118 (54.5)	299,254 (54.0)	577,864 (54.8)
Urban	731,422 (45.5)	254,555 (46.0)	476,867 (45.2)
Household income, *n* (%)			
High (70−100th percentile)	684,264 (42.5)	248,078 (44.8)	436,186 (41.4)
Middle (30−69th percentile)	660,916 (41.1)	220,050 (39.7)	440,866 (41.8)
Low (0−29th percentile)	263,360 (16.4)	85,681 (15.5)	177,679 (16.9)
Preterm birth, ≤36 week, *n* (%)	33,294 (2.1)	655 (0.1)	32,639 (3.1)
Low birth weight, ≤2499 g, *n* (%)	26,001 (1.6)	428 (0.1)	25,573 (2.4)
Types of infant feeding			
Fully formula feeding	626,656 (39.0)	197,850 (35.7)	428,806 (40.7)
Partially breastfeeding	325,581 (20.2)	113,833 (20.6)	211,748 (20.1)
Exclusive breastfeeding	656,303 (40.8)	242,126 (43.7)	414,177 (39.3)

### Breastfeeding and overall hospital admissions

When compared to children with fully formula feeding, the hospitalization rate was 12% lower in those with partially breastfeeding (adjusted incidence rate ratio [IRR], 0.88; 95% confidence interval [CI], 0.88 to 0.89) and 15% lower in those with exclusive breastfeeding (adjusted IRR, 0.85; 95% CI, 0.85 to 0.86). The hospital admission rate (100 person-years) was: 25.15 for fully formula feeding vs. 21.78 for partially breastfeeding vs. 20.30 for exclusive breastfeeding. Similar effect sizes and patterns of hospital admission rates were found in all different groups following stratification by sex, birth season, and region of residence (Table 2). As the participants grew older, the protective effect on hospitalization of exclusive breastfeeding gradually decreased (Table 3; 18%, 17%, 13%, 12%, and 14% lower in those with exclusive breastfeeding aged <1, 1−2, 3−4, 5−6, and 7−10 years, respectively, compared to those with fully formula feeding).

**Table 2:** Statistical model to determine the relationship between types of infant feeding and overall hospital admissions (unmatched cohort; *n* = 1,608,540)

Parameter	*n* (%)	Admission events (individuals with at least one event)	Person-years	Incidence rate of admission events^a^	Incidence rate ratio of admission events (95% CI)	
					Sex-adjusted	Adjusted^b^
Feeding types (overall)						
Fully formula feeding	626,656 (39.0)	1,245,224 (428,589)	4,952,136.6	25.15	1.0 (reference)	1.0 (reference)
Partially breastfeeding	325,581 (20.2)	564,097 (211,617)	2,590,452.2	21.78	**0.87 (0.87−0.88)**	**0.88 (0.88−0.89)**
Exclusive breastfeeding	656,303 (40.8)	1,086,482 (413,815)	5,350,983.4	20.30	**0.83 (0.83−0.84)**	**0.85 (0.85−0.86)**
Male						
Fully formula feeding	323,878 (40.1)	678,750 (228,573)	2,561,925.8	26.49	1.0 (reference)	1.0 (reference)
Partially breastfeeding	171,923 (21.3)	316,118 (115,663)	1,370,077.7	23.07	**0.88 (0.87−0.88)**	**0.89 (0.88−0.90)**
Exclusive breastfeeding	312,800 (38.7)	549,216 (204,515)	2,555,983.3	21.49	**0.84 (0.83−0.84)**	**0.85 (0.85−0.86)**
Female						
Fully formula feeding	302,778 (37.9)	566,474 (200,016)	2,390,210.8	23.70	1.0 (reference)	1.0 (reference)
Partially breastfeeding	153,658 (19.2)	247,979 (95,954)	1,220,374.4	20.32	**0.86 (0.86−0.87)**	**0.88 (0.87−0.88)**
Exclusive breastfeeding	343,503 (42.9)	537,266 (209,300)	2,795,000.1	19.22	**0.84 (0.83−0.84)**	**0.85 (0.85−0.86)**
Calendar period of birth (2009-2010)						
Fully formula feeding	217,864 (36.4)	438,412 (147,813)	2,137,191.2	20.51	1.0 (reference)	1.0 (reference)
Partially breastfeeding	116,632 (19.5)	207,350 (75,914)	1,143,333.6	18.14	**0.88 (0.88−0.89)**	**0.89 (0.89−0.90)**
Exclusive breastfeeding	264,781 (44.2)	444,335 (166,816)	2,598,500.9	17.10	**0.83 (0.83−0.84)**	**0.855 (0.85−0.86)**
Calendar period of birth (2011-2012)						
Fully formula feeding	189,611 (38.6)	386,770 (130,812)	1,512,973.5	25.56	1.0 (reference)	1.0 (reference)
Partially breastfeeding	99,962 (20.4)	175,864 (65,290)	796,665.3	22.08	**0.86 (0.85−0.87)**	**0.87 (0.87−0.88)**
Exclusive breastfeeding	201,416 (41.0)	339,580 (127,887)	1,610,556.1	21.08	**0.83 (0.82−0.83)**	**0.85 (0.84−0.86)**
Calendar period of birth (2013-2015)						
Fully formula feeding	219,181 (42.3)	420,042 (149,964)	1,301,971.9	32.26	1.0 (reference)	1.0 (reference)
Partially breastfeeding	108,987 (21.0)	180,883 (70,413)	650,453.3	27.81	**0.87 (0.86−0.87)**	**0.88 (0.87−0.89)**
Exclusive breastfeeding	190,106 (36.7)	302,567 (119,112)	1,141,926.4	26.50	**0.83 (0.82−0.84)**	**0.85 (0.85−0.86)**
Rural						
Fully formula feeding	349,079 (39.8)	708,624 (239,763)	2,747,437.1	25.79	1.0 (reference)	1.0 (reference)
Partially breastfeeding	170,608 (19.5)	302,714 (111,209)	1,353,312.6	22.37	**0.87 (0.87−0.88)**	**0.88 (0.88−0.89)**
Exclusive breastfeeding	357,431 (40.8)	602,535 (226,569)	2,899,147.4	20.78	**0.83 (0.83−0.84)**	**0.85 (0.84−0.85)**
Urban						
Fully formula feeding	277,577 (38.0)	536,600 (188,826)	2,204,699.4	24.34	1.0 (reference)	1.0 (reference)
Partially breastfeeding	154,973 (21.2)	261,383 (100,408)	1,237,139.5	21.13	**0.87 (0.87−0.88)**	**0.88 (0.88−0.89)**
Exclusive breastfeeding	298,872 (40.9)	483,947 (187,246)	2,451,836.0	19.74	**0.84 (0.83−0.84)**	**0.86 (0.85−0.86)**

We performed the negative binomial regression model (endpoint, incidence rate of any hospital admission) with incidence rate ratios and 95% CIs.

Numbers in bold indicate significant differences (two-sided *p* <  0.05).

*CI* confidence interval.

^a^Incidence rate of admission events is expressed as per 100 person-years.

^b^Adjusted model: adjusted for infant sex, calendar period of birth (2009−2010, 2011−2012, and 2013−2015), birth season (spring, summer, autumn, and winter), region of residence (rural and urban), household income (high, middle, and low), preterm birth, and low birth weight.

**Table 3:** Hospital admissions during childhood by types of infant feeding, stratified by age at admission (unmatched cohort; *n* = 1,608,540)

Age at admission	Types of infant feeding		
	Fully formula feeding (*n* = 626,656)	Partially breastfeeding (*n* = 325,581)	Exclusive breastfeeding (*n* = 656,303)
<1 year, incidence rate of admission events^b^	63.36	55.09	50.90
Sex-adjusted IRR (95% CI)	1.0 (reference)	**0.87 (0.86−0.88)**	**0.80 (0.80−0.81)**
Adjusted IRR^a^ (95% CI)	1.0 (reference)	**0.89 (0.89−0.90)**	**0.82 (0.82−0.83)**
1−2 years, incidence rate of admission events^b^	34.51	29.59	28.07
Sex-adjusted IRR (95% CI)	1.0 (reference)	**0.86 (0.85−0.86)**	**0.81 (0.81−0.82)**
Adjusted IRR^a^ (95% CI)	1.0 (reference)	**0.87 (0.86−0.88)**	**0.83 (0.83−0.84)**
3−4 years, incidence rate of admission events^b^	17.49	15.48	14.99
Sex-adjusted IRR (95% CI)	1.0 (reference)	**0.89 (0.88−0.89)**	**0.86 (0.85−0.87)**
Adjusted IRR^a^ (95% CI)	1.0 (reference)	**0.90 (0.89−0.91)**	**0.87 (0.87−0.88)**
5−6 years, incidence rate of admission events^b^	12.38	10.85	10.44
Sex-adjusted IRR (95% CI)	1.0 (reference)	**0.89 (0.88−0.90)**	**0.89 (0.88−0.90)**
Adjusted IRR^a^ (95% CI)	1.0 (reference)	**0.89 (0.88−0.91)**	**0.88 (0.87−0.88)**
7−10 years, incidence rate of admission events^b^	8.81	7.76	7.40
Sex-adjusted IRR (95% CI)	1.0 (reference)	**0.95 (0.94−0.96)**	**0.90 (0.89−0.92)**
Adjusted IRR^a^ (95% CI)	1.0 (reference)	**0.89 (0.87−0.90)**	**0.86 (0.85−0.87)**

We performed the negative binomial regression model (endpoint, incidence rate of any hospital admission) with incidence rate ratios and 95% CIs.Numbers in bold indicate significant differences (two-sided *p*  <  0.05).

*IRR* incidence rate ratio, *CI* confidence interval.

^a^Adjusted model: adjusted for infant sex, calendar period of birth (2009−2010, 2011−2012, and 2013−2015), birth season (spring, summer, autumn, and winter), region of residence (rural and urban), household income (high, middle, and low), preterm birth, and low birth weight.

^b^Incidence rate of admission events is expressed as per 100 person-years.

### Breastfeeding and hospital admissions by cause of morbidity

**Fig. 3:**
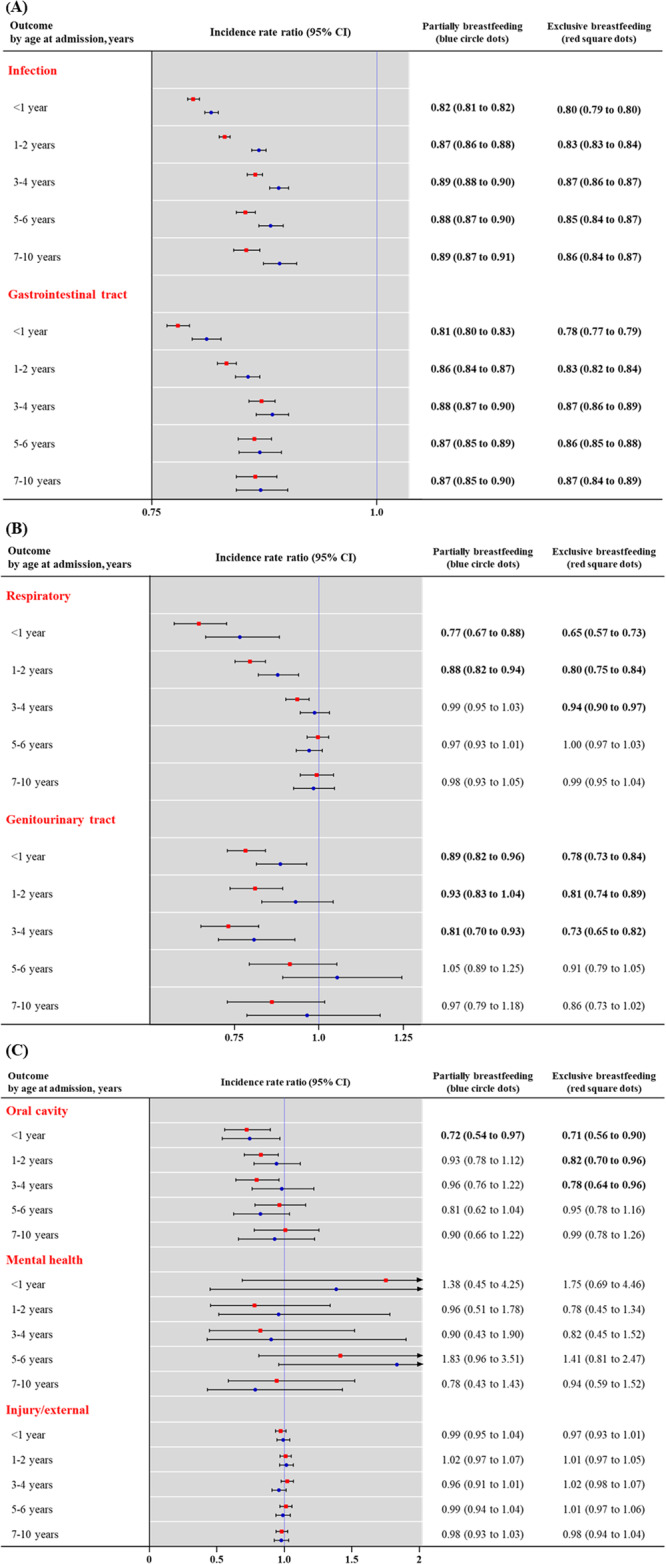
Incidence rate ratio of hospital admissions by types of infant feeding, stratified by age at admission and cause of morbidity. (**A**) infection and gastrointestinal tract; (**B**) respiratory and genitourinary tract; and (**C**) oral cavity, mental health, and injury/external. Incidence rate ratio (blue circle and red square dots) with 95% CI (error bar) for key causes of infection, gastrointestinal tract, respiratory, genitourinary tract, oral cavity, mental health, and injury/external. We performed the negative binomial regression model (endpoint, incidence rate of any hospital admission) with incidence rate ratios and 95% CIs. Models were adjusted for infant sex, calendar period of birth (2009−2010, 2011−2012, and 2013−2015), birth season (spring, summer, autumn, or winter), region of residence (rural or urban), household income (high, middle, or low), preterm birth (≤36 weeks), and low birth weight (≤2499 g). Numbers in bold indicate significant differences (two-sided *p* < 0.05).*CI* confidence interval. Source data are provided as a Source Data file.

Figure 3 shows adjusted IRR with 95% CI for key causes of morbidity (oral cavity, injury/external, mental health, infection, gastrointestinal tract, respiratory, and genitourinary tract) by feeding type and age at admission (<1, 1−2, 3−4, 5−6, and 7−10 years). There were no increased risks of hospitalization associated with mental health and injury/external cause. The highest risk of hospitalization was due to ailments of the oral cavity, gastrointestinal tract, respiratory, and genitourinary tract, and infection during infancy, and the risk of hospitalization declined with age. These excess risks remained until 3−4 years (respiratory, genitourinary, and oral cavity), and persisted until 10 years (gastrointestinal tract and infection).

### Sensitivity analysis

To determine the robustness and generalization of our main findings, we performed an additional sensitivity analysis. First, we generated a 1:1 exposure-driven propensity score-matched cohort of two groups of children with fully formula feeding vs. exclusive breastfeeding (*n* = 600,988 for each group) to strengthen and generalize our main results. After a 1:1 exposure-driven propensity score matching, there were no major imbalances in the baseline sociodemographic characteristics evaluated using standardized mean differences (SMDs; all SMDs < 0.07). The results of the sensitivity analysis in the matched cohort are also consistent with our main results (Supplementary Tables 3−7).

## Discussion

### Principal findings

In this Korean nationwide birth cohort study, breastfeeding for at least 6 months was associated with a lower risk for subsequent hospital admissions, and this lower risk was evident in the exclusive breastfeeding group. The magnitude of the protective effect of breastfeeding on hospitalization decreased with age. These decreased risks of subsequent hospital admission remained significant until the child was 3−4 years old for non-infection respiratory, oral cavity, and non-infection genitourinary tract and 10 years for infection and non-infection gastrointestinal tract. However, breastfeeding did not reduce the risk of hospitalization due to mental health and injury. Although the analysis conditions were changed several times, patterns and associations of our main result showed consistency.

### Comparison with other studies

Although mounting studies have investigated the protective effect of breastfeeding for subsequent hospital admission, there is a paucity of literature that has used a comprehensive approach. Further, the study designs of previous studies are not robust to investigate the protective effect of breastfeeding and attenuated time effect stratified by cause of morbidity^9–15^.

Previous studies on breastfeeding and the risk of subsequent hospital admission have shown a beneficial associations in the United Kingdom (*n* = 502,948; infection, allergic disorders, diabetes, and dental caries^9^ or *n* = 15,890; respiratory and gastrointestinal tract infections^10^), Australia (*n* = 2602; respiratory diseases)^11^, Spain (*n* = 1385; infection and gastrointestinal tract diseases)^12^, Italy (*n* = 460; fever)^13^, China (*n* = 678; fever)^14^, and India (*n* = 232, infection)^15^, which is in well correspondence with our main findings. However, small sample sizes and insufficient statistical power, heterogeneous study population or single hospital-based studies, short-term follow-up period, uncontrolled study design (i.e., cross-sectional or uncontrolled cohort design), lack of comprehensive approach with a special focus on infection-related admission, or a lack of consideration for the attenuation in protective effects over time contributing to low levels of evidence and uncertain results^9–15^. With our comprehensive approach, we provide evidence that though the effect of breastfeeding was reduced over time, the protective effect of breastfeeding on infections persisted until 10 years old. Although it was shorter than for infection, breastfeeding also showed protective effect on other causes of disease (i.e., oral cavity, non-infection genitourinary tract, non-infection respiratory, and non-infection gastrointestinal tract).

In addition to the nutritional and physical health benefits of breastfeeding, several previous studies have shown that breastfeeding has significant and far-reaching effects on cognition, mental health, and behavior in children^17,18^. However, our results showed that the risks of hospitalization due to mental health problems were not significantly affected by breastfeeding. These negative outcomes may be due to fewer hospitalizations for cognitive and behavioral disorders, where breastfeeding is known to be beneficial for children. In addition, the rate of admission to a psychiatric ward for children under the age of ten is relatively low^19^. In another study, the most common causes of hospitalization in children were depression, bipolar disorder, and psychosis, and only 11.9% of hospitalized children were younger than 10 years of age^19^. Although breastfeeding may have protective effects on cognition, mental health, and behavior, our results showed that in children, breastfeeding is not associated with a reduced risk of hospitalization for serious psychiatric disorders, such as psychosis.

### Plausible mechanism

The hospitalization rate for each reason decreased with age, and the duration of breastfeeding effect was different depending on the cause of morbidity. First, increasing evidence suggests that infant nutrition impact the infant microbiome trajectory and immune competence development. The feeding mode, exclusive or partial breastfeeding vs no breastfeeding during infancy, has well-recognized effects on the gut microbiome composition and function^20^. Therefore, the health benefits observed in breastfed infants may partly be explained by a more appropriate gut microbiome maturation. Differently from adult, the intestinal T cell compartment in neonates is characterized by high levels of suppressive regulatory T cells to control immune responses and maintain gut immune homeostasis^21^. Since microbiome-host immune system interactions in early life dictate long-term immune functionality^22^ it is very important that the ‘right’ symbionts colonize the intestine at the ‘right’ time^23^. Therefore, changes in the gut microbiome of infants caused by breastfeeding lead to an appropriate immune system interaction and are thought to affect immunity in later life and consequent hospitalization rate. Second, in respiratory tract problems including asthma and other allergic disease that result from an inappropriate Th2 response to environmental antigen, breastfeeding-induced inhibition of Th2 immune responses in rodents fostered by antigen-sensitized mothers has been extensively studied^24–26^. The proposed mechanism was that it was dependent on the induction of antigen-specific IgG following maternal immunization and their transfer to the neonates through breast milk. Third, an in vitro study reported an important role of breast milk soluble CD14 in B cell growth and differentiation^26^. Although the function is not known yet, it may play a role in colonization of the gastrointestinal tract, and as a result in the generation of an atopic phenotype^27^. A protective synergistic effect of soluble CD14 levels and breastfeeding on the risk of asthma was reported in a large prospective cohort study, especially in children of mothers without a history of atopic disease^28^. Fourth, the presence of IgA in human breast milk is well documented. Maternal pathogen-specific IgAs in breast milk are crucial in preventing respiratory and gut infections in breastfed children^29^.

The reason for the different duration of hospitalization reduction effect of breast milk for each reason could not be clearly identified from the results of this study. Perhaps it is because the duration and change of microbiomes derived from breast milk over time are different for each organ. For example, in the oral cavity, breastmilk plays a prebiotic role in the selection of early-colonizing, health-associated oral bacteria, such as *Streptococcus mitis group*. These oral microbiomes became more heterogeneous following the introduction of solid foods^29,30^.

### Policy implications

Overall, the findings of our study emphasize the need for strategies aimed at preventing and managing hospital admission in infants and children. The WHO and UNICEF recommend EBF-6 with continued breastfeeding until 2 years and beyond^9–15^. However, mothers encounter difficulties in breastfeeding and may have other challenges^31^. The reasons to stop or avoid breastfeeding range from medical, cultural, and psychological or physical discomfort and inconvenience^31^. These issues are magnified across populations, and with the involvement of multinational commercial interests, this situation has catastrophic consequences on breastfeeding rates and the health of subsequent generations^9–15^. More importantly, genuine and urgent commitment is crucial from governments and health authorities to establish a new normal: where every woman understands the importance of breastfeeding, expects to do it, and receives all possible supports for it. There are possible concerns regarding how this recommendation may be unsuitable for certain subgroups of infants (i.e., those born with low iron stores, at high risk of developing food allergies, or not being able to consume an adequate volume of milk) that needs to be carefully designed as a part of guidelines. In such cases, education programs for parents that are tailored to support specific nutritional needs would be essential.

### Strengths and limitations

When interpreting our main results, several limitations should be considered. First, data on types of infant feeding was based on reported data by parents. This may lead to misclassification due to recall bias, regression dilution bias, and underestimation of the magnitude of true association^32^. However, in Korea, physicians do interact with the parents using the questionnaire during medical consultations, which may reduce this concern. Second, the data on types of infant feeding was obtained at a single point of a nationwide health examination in infants aged 6 months. This single-point examination can lead to an overestimate of breastfeeding than average with variations in possible effects and a longer period of recall bias. Further, it is difficult to verify the long-term effect of breast milk intake over 6 months. Third, although we performed an adjusted propensity score matching model using various confounding factors, it is important to acknowledge that since the data is observational, we cannot rule out the possibility of residual confounding due to unmeasured factors. A large-scale interventional trial to investigate the true magnitude of the relationship between breastfeeding and any admission in children would add more insights. Fourth, we enrolled only infants who received the first National Health Screening Program for Infants and Children and could only assess the data that consisted of approximately 80% of national births^33^. To address the heterogeneity of our population, we provided the table of representativeness of the study participants (Supplementary Table 1)^34^. As compared to other representative national surveys in Korea that report 31.8% breastfeeding rate, 40.8% breastfeeding rate reported in our study was reliable^35^. Fifth, our study population was limited to only Koreans, and we should be cautious in interpreting and generalizing our main findings in other ethnic groups. Sixth, though we performed a sophisticated statistical technique through a large-scale nationwide birth cohort to reduce several biases, scanty parental investment associated with formula feeding can cause more frequent infection and admission rates in offspring that may inadvertently affect the true magnitude of association between breastfeeding and subsequent hospital admission in children. Finally, a defined mechanism for the differences in hospitalizations rates and causes of hospitalization by age could not be elucidated.

Despite these limitations, our large-scale, population-based, nationwide birth cohort study investigated the potential association between breastfeeding and subsequent hospital admission in children. We used a large representative sample (1.61 million children) and sophisticated statistical techniques to strengthen and generalize our main findings. This is the largest analysis to evaluate the protective breastfeeding effect of subsequent hospital admission and the first to focus on the lag time effect of subsequent hospital admission. Overall, the findings of this study have illustrated the need for strategies aimed at the prevention and management of any admission in children.

In conclusion, our nationwide birth cohort study in Korea provides evidence that breastfeeding for 6 months was associated with a lower risk for subsequent hospital admission, and this lower risk was particularly pronounced for exclusive breastfeeding. In particular, the magnitude of the protective effect of breastfeeding declined with age. Interestingly, these attenuated risks of subsequent hospital admission remained significant until 3−4 years (non-infection respiratory, oral cavity, and non-infection genitourinary tract) and persisted until 10 years (infection and non-infection gastrointestinal tract). However, breastfeeding did not reduce the risk of hospitalization due to mental health and injury. Patterns and associations of the sensitivity analyses were consistently similar to our main results, suggesting that our findings are robust. The findings of the study suggest the need for tailored strategies aimed at the prevention and management of any admission in children. Encouraging mothers to maintain breastfeeding for the first 6 months of life in infants should be a key public health strategy.

## Methods

### Data source

This study was based on the National Health Insurance Service in the Republic of Korea. We selected the dataset of all infants born between 1 January 2009 and 31 December 2015, and who had records of receiving the first National Health Screening Program for Infants and Children. According to birth statistics, our birth cohort covered approximately 80% of national births^33^. The dataset was linked and consisted of data on first general health examination results, death records, health insurance data including insurance eligibility data, personal sociodemographic data, inpatient and outpatient healthcare records, and medication records. The data were anonymized to ensure the confidentiality required by the South Korean government^36^. The study was approved by the Institutional Review Board of Sejong University (Seoul, South Korea; SJU-HR-E-2021-001) and Seoul National University (Seoul, South Korea; E-2108-134-1246). The Korean National Health Insurance Service and the Korean Government provided information governance approval (NHIS-2022-1-383). Under the terms of the approval, patient consent was not required for use of routine health records for our study.

### Study design and participants

The study was a population-based, nationwide birth cohort study consisting of all Korean infants born between 1 January 2009 and 31 December 2015, and who received the first National Health Screening Program for Infants and Children (*n* = 2,010,325). The Korean government provides a complimentary first general health examination to all Korean infants aged 6 months. Among 2,010,325 infants in South Korea, we excluded infants (*n* = 401,785) diagnosed with a congenital anomaly in the first 6 months of life and those diagnosed with malignant neoplasm, a blood disorder, chronic kidney disease, cystic fibrosis, and/or immune dysfunction. The final sample size was 1,608,540 infants (Figs. 1, 2). The observation period was between 1 January 2009 and 31 December 2019. The date of the first National Health Screening Program for Infants and Children was used as the date of entry in cohort.

Calendar period of birth, sex, birth season, region of residence, and household income were obtained from insurance eligibility and personal data. Preterm birth and low birth weight were obtained from the first National Health Screening Program for Infants and Children, including self-reported questionnaires and personal medical interviews with their parents.

### Exposures and outcome

Type of infant feeding was documented during the first general health examination to all Korean infants aged 6 months. Parents of the participating infants were asked to report the types of infant feeding in three categories: exclusive breastfeeding, partially breastfeeding, and fully formula feeding.

The primary outcome was the incidence rate of any hospital admission during the observation period between 1 January 2009 and 31 December 2019. To reduce reverse causation, primary outcome was defined only as hospital admission after first general health examination. We reported the following stratified periods of follow-up: less than 1 year, 1−2 years, 3−4 years, 5−6 years, and 7−10 years^37^. The secondary outcomes were the number of hospital admissions by cause of morbidity (infection, non-infection respiratory, non-infection gastrointestinal tract, non-infection genitourinary tract, non-infection oral cavity, mental health, and injury/external). We used the primary diagnosis code within each admission to define the cause of each admission, grouped as per the International Classification of Diseases, tenth revision (ICD-10) codes (Supplementary Table 8)^37^.

### Statistical analysis

Supplementary Table 1 gives a justification for the selected indicators and chosen study population^34^. In our birth cohort, “exposure” was the type of infant feeding (fully formula feeding, partially breastfeeding, and exclusive breastfeeding), “primary outcome” was the incidence of any hospital admission during observation period, “secondary outcomes” were the number of hospital admissions by cause of morbidity, and “individual index date” was the date of the first general health examination. The follow-up ended on 31 December 2019 or at death. The hospital admission rate was expressed per 100 person-years. Statistical analyses were performed using R software (version 3.1.1; R Foundation, Vienna, Austria) for generating figures, Statistical Package for Social Sciences (version 25.0; IBM Corp, Armonk, NY, USA) for exploratory data analysis, and SAS (version 9.4; SAS Institute Inc., Cary, NC, USA) for big-data analysis. A two-sided *p* value of less than 0.05 was considered statistically significant.

We performed the negative binomial regression model (endpoint, incidence rate of any hospital admission) with IRRs and 95% confidence intervals (CIs)^38–40^. Models were adjusted for infant sex, calendar period of birth (2009−2010, 2011−2012, and 2013−2015), birth season (spring, summer, autumn, or winter), region of residence (rural or urban)^32,33,41^, household income (high, middle, or low), preterm birth (≤36 weeks), and low birth weight (≤2499 g).

### Subgroup and sensitivity analysis

Various subgroup and sensitivity analyses were performed:^42^ a stratification analysis based on hospital admission risk at different time points (less than 1 year, 1−2 years, 3−4 years, 5−6 years, and 7−10 years) or on cause of morbidity (infection, non-infection respiratory, non-infection gastrointestinal tract, non-infection genitourinary tract, non-infection oral cavity, mental health, and injury/external); a subgroup analysis according to infant sex, calendar period of birth, and region of residence; and alternative cohort specifications (propensity-score-matched cohort). To investigate potential heterogeneity in the effects of breastfeeding on hospitalization outcomes, we have decided to conduct three additional subgroup analyses (age, calendar period of birth, and region of residence) based on existing literature or biological hypothesis^2,43,44^.

We used a propensity score-matched cohort to determine robustness and generalization of our main results^41^. We performed 1:1 exposure-driven propensity score matching to balance the baseline covariates of two groups to minimize potential confounding effects. Propensity scores were derived using a multivariable logistic regression model from the predicted probability of exclusively breastfed infants vs. fully formula-fed infants (each *n* = 600,988). The following covariates were considered potential confounding factors: infant sex, calendar period of birth, birth season, region of residence, household income, preterm birth, and low birth weight. A nearest-neighbor algorithm was used to match infants in two groups with a random selection without replacement within specified caliper widths (0.001 standard deviations). The adequacy of exposure-driven propensity score matching was assessed by comparing SMDs^41^. We considered that an SMD of less than 0.1 indicated a lack of major imbalance in two groups^32,41^. This study followed the Strengthening the Reporting of Observational Studies in Epidemiology (STROBE) reporting guideline (Supplementary Table 9).

### Patient and public involvement

Patients or their parents were not involved in setting the research question or the outcome measures, nor were they involved in the design and implementation of the study.

### Reporting summary

Further information on research design is available in the Nature Portfolio Reporting Summary linked to this article.

### Supplementary information

Supplementary Information File

Reporting Summary

### Source data

Source Data

## Data Availability

The datasets analysed during the current study are available in the National Health Insurance Service, South Korea, https://nhiss.nhis.or.kr/bd/ab/bdaba000eng.do. This protects the confidentiality of the data and ensures that Information Governance is robust. Applications to access health data in South Korea are submitted to the National Health Insurance Service, South Korea. Information can be found at https://nhiss.nhis.or.kr/bd/ab/bdaba000eng.do. Source data (Fig. 3) are provided with this paper. Source data are provided with this paper.

## Code availability

This study did not generate new or customized code/algorithm. Statistical analyses were performed using R software (version 3.1.1; R Foundation, Vienna, Austria) for generating figures, Statistical Package for Social Sciences (version 25.0; IBM Corp, Armonk, NY, USA) for exploratory data analysis, and SAS (version 9.4; SAS Institute Inc., Cary, NC, USA) for big-data analysis. The codes used in the analysis are available from the corresponding author.
