# Supplementary material for: Breastfeeding and impact on childhood hospital admissions: a nationwide birth cohort in South Korea
Source: Nat Commun. 2023 Sep 20;14:5819. doi: 10.1038/s41467-023-41516-y (PMC10511528; doi:10.1038/s41467-023-41516-y)
Supplement: Supplementary file 1 — Supplementary Information File [file 41467_2023_41516_MOESM1_ESM.pdf]

Supplementary information

Breastfeeding and impact on childhood hospital admissions: a nationwide birth cohort in South Korea

Jeong-Seon Lee, MD,[1] Jae Il Shin, MD, PhD,[2] Sunyeup Kim, BS,[3] Yong Sung Choi, MD, PhD,[4¶] Youn Ho Shin, MD, PhD,[5] Jimin Hwang, MD,[6] Jung U Shin, MD, PhD,[7] Ai Koyanagi, MD, PhD,[8] Louis Jacob, MD, PhD,[9] Lee Smith, PhD,[10] Han Eol Jeong, PhD, MPH,[11] Yunha Noh, PharmD, PhD,[11,19,20] In-Sun Oh, PhD,[11,19,20] Sang Youl Rhee, MD,[12] Chanyang Min, PhD,[12] Seong Ho Cho, MD,[13] Steve Turner, MD,[14] Guillaume Fond, MD, PhD,[15] Laurent Boyer, MD, PhD,[15] Dong In Suh, MD,[16] Krishna Prasad Acharya, MD,[17] Ju-Young Shin, PhD,[11,18] Seung Won Lee, MD, PhD,[3] Dong Keon Yon, MD, PhD[4,12]

[1]Department of Pediatrics, Soonchunhyang University Bucheon Hospital, Soonchunhyang University School of Medicine, Bucheon, Republic of Korea.

[2]Department of Pediatrics, Yonsei University College of Medicine, Seoul, Republic of Korea.

[3]Department of Precision Medicine, Sungkyunkwan University School of Medicine, Suwon, Republic of Korea.

[4]Department of Pediatrics, Kyung Hee University Medical Center, Kyung Hee University College of Medicine, Seoul, Republic of Korea.

[5]Department of Pediatrics, The Catholic University of Korea, Yeouido St. Mary's Hospital, Seoul, Republic of Korea.

[6]Department of Epidemiology, Johns Hopkins Bloomberg School of Public Health, Baltimore,

MD, USA.

[7]Department of Dermatology, CHA Bundang Medical Center, CHA University School of Medicine, Seongnam, Republic of Korea.

[8]Research and Development Unit, Parc Sanitari Sant Joan de Déu, CIBERSAM, ISCIII, Barcelona, Spain.

[9]Department of Physical Medicine and Rehabilitation, Lariboisière-Fernand Widal Hospital, AP-HP, Université Paris Cité, Paris, France.

[10]Centre for Health, Performance and Wellbeing, Anglia Ruskin University, Cambridge, UK.

[11]School of Pharmacy, Sungkyunkwan University, Suwon, Republic of Korea.

[12]Center for Digital Health, Medical Science Research Institute, Kyung Hee University Medical Center, Kyung Hee University College of Medicine, Seoul, Republic of Korea.

[13]Division of Allergy-Immunology, University of South Florida Morsani College of Medicine, Tampa, FL, USA.

[14]Maternity and Child Health Division, NHS Grampian, Aberdeen, UK.

[15]CEReSS-Health Service Research and Quality of Life Center, Assistance Publique-Hopitaux de Marseille, Aix-Marseille University, Marseille, France.

[16]Department of Pediatrics, Seoul National University Hospital, Seoul National University College of Medicine, Seoul, Republic of Korea.

[17]Animal Quarantine Office Kathmandu, Budhanilkantha, Kathmandu, Nepal.

[18]Department of Biohealth Regulatory Science, Sungkyunkwan University, Suwon, Republic of Korea.

[19]Deparments of Epidemiology, Biostatistics, and Occupational Health, McGill University, Montreal, Canada

[20]Centre for Clinical Epidemiology, Lady Davis Institute, Jewish General Hospital, Montreal, Canada

These authors contributed equally: Jeong-Seon Lee, Jae Il Shin, Sunyeup Kim, Yong Sung Choi.

These authors jointly supervised this work: Ju-Young Shin, Seung Won Lee, Dong Keon Yon.

Supplementary Table 1 | Representativeness and justification of study participants

Category	Justification
Disease	Any hospital admission [infection, non-infection respiratory, non-infection gastrointestinal tract, non-infection genitourinary tract, non-infection oral cavity, mental health, and injury (external)]
Special considerations related to	
Sex	The hospital admission rate affects boys more than girls. Therefore, we performed additional stratification analysis.
Age	The hospital admission rate in children is lower as age increases.
Socioeconomic status	Socioeconomic status is an important determinant affecting the hospital admission rate.
Race	The hospital admission rate affects all races across the world with different social environment status among races.
Geography of urban-rural interaction	Environmental factors may have a different impact on all health outcomes in urban and rural communities. Therefore, we performed additional stratification analysis.
Other considerations	Throughout the world, hospital admissions vary widely within and among countries. They are associated with major comorbidities, including congenital anomalies, birth season, preterm birth, and low birth weight.
Overall representativeness of this study	We used a large representative sample (1.61 million children) and sophisticated statistical techniques to strengthen and generalize our main findings. This is largest analysis to evaluate protective breastfeeding effect of subsequent hospital admission and first analysis to focus on the lag time effect of subsequent hospital admission. We should determine whether the effect differs depending on when the individuals were born. Within each cohort, we compared the incidence rates of the primary outcome on those who were followed up for various periods (calendar period of birth).

Supplementary Table 2 | Distribution of hospital admissions by types of infant feeding, stratified by age at admission (unmatched cohort; *n* = 1,608,540)

Parameter	Exclusive breastfeeding		Partially breastfeeding		Fully formula feeding	
	Admission events	Individuals with at least one event	Admission events	Individuals with at least one event	Admission events	Individuals with at least one event
Overall hospital admissions						
Overall	413,815	1,086,482	211,617	564,097	428,589	1,245,224
<1 year	243,932	334,044	129,612	179,361	271,070	397,013
1-2 years	201,103	368,362	103,237	192,613	219,940	432,391
3-4 years	125,540	196,642	63,633	100,685	132,930	218,978
5-6 years	84,229	117,354	41,884	58,504	87,726	126,453
7-10 years	49,344	70,080	22,903	32,934	47,688	70,389
Infection						
Overall	302,304	702,287	153,977	362,889	323,451	819,899
<1 year	117,877	154,390	60,928	80,637	138,784	193,444
1-2 years	169,892	287,920	87,842	151,144	188,669	339,843
3-4 years	96,284	141,308	49,105	72,594	103,320	158,604
5-6 years	58,006	76,513	29,211	38,529	62,256	84,509
7-10 years	31,897	42,156	15,106	19,985	31,993	43,499
Gastrointestinal tract						
Overall	113,930	168,996	57,030	85,136	124,745	194,507
<1 year	27,821	31,328	14,506	16,442	34,312	39,793
1-2 years	51,130	64,911	25,856	33,113	57,720	75,735
3-4 years	30,637	37,025	15,357	18,642	33,322	41,219
5-6 years	18,349	21,346	9,009	10,386	19,590	23,150
7-10 years	12,013	14,386	5,413	6,553	11,866	14,610
Respiratory						
Overall	19,556	20,747	9,805	10,434	19,559	20,973
<1 year	460	470	279	285	702	734
1-2 years	2,350	2,481	1,296	1,380	2,867	3,076
3-4 years	5,842	6,080	3,127	3,236	6,078	6,325
5-6 years	7,885	8,080	3,773	3,875	7,438	7,655
7-10 years	3,521	3,636	1,592	1,658	3,067	3,183
Genitourinary tract						
Overall	3,394	3,661	1,947	2,129	4,103	4,496
<1 year	1,381	1,449	826	867	1,795	1,880
1-2 years	800	834	468	500	974	1,029
3-4 years	546	578	310	328	701	776
5-6 years	415	449	229	254	433	461
7-10 years	321	351	155	180	307	350
Oral cavity						
Overall	947	978	483	496	1,079	1,107
<1 year	117	119	62	62	169	170
1-2 years	309	314	172	174	363	370
3-4 years	175	178	106	109	216	220
5-6 years	210	214	84	88	208	214
7-10 years	146	153	62	63	131	133
Mental health						
Overall	158	343	80	185	203	289
<1 year	11	11	5	5	8	8
1-2 years	26	26	13	17	33	33
3-4 years	26	49	12	37	47	58
5-6 years	43	99	22	60	51	59
7-10 years	57	158	34	66	65	131
Injury/External						
Overall	23,101	26,014	11,280	12,648	21,845	24,604
<1 year	4,925	5,065	2,559	2,652	5,026	5,231
1-2 years	5,032	5,490	2,538	2,757	4,845	5,250
3-4 years	4,444	4,930	2,132	2,312	4,214	4,630
5-6 years	4,555	5,065	2,163	2,425	4,208	4,667
7-10 years	4,824	5,464	2,200	2,502	4,187	4,826

Supplementary Table 3 | Sociodemographic characteristics of participants in the 1:1 propensity-score-matched cohort (n = 1,201,976)

Characteristic	Full unmatched cohort (total)	1:1 propensity-score-matched cohort		SMD[a]
		Exclusive breastfeeding	Fully formula feeding	
Total, *n* (%)	1,608,540 (100.0)	600,988	600,988	
**Baseline characteristics**				
Infant sex, *n* (%)				0.069
Female	799,939 (49.7)	285,232 (47.5)	305,844 (50.9)	
Male	808,601 (50.3)	315,756 (52.5)	295,144 (49.1)	
Calendar period of birth, *n* (%)				0.09
2008-2010	599,277 (37.3)	239,845 (39.9)	214,081 (35.6)	
2011-2012	490,989 (30.5)	176,765 (29.4)	185,008 (30.8)	
2013-2015	518,274 (32.2)	184,378 (30.7)	201,899 (33.6)	
Birth season				0.058
Spring (March−May)	401,371 (25.0)	156,064 (26.0)	150,835 (25.1)	
Summer (June−August)	362,642 (22.5)	145,722 (24.2)	135,158 (22.5)	
Autumn (September−November)	416,415 (25.9)	143,334 (23.8)	155,050 (25.8)	
Winter (December−February)	428,112 (26.6)	155,868 (25.9)	159,945 (26.6)	
Region of residence, *n* (%)				
Rural	877,118 (54.5)	341,412 (56.8)	332,474 (55.3)	0.03
Urban	731,422 (45.5)	259,576 (43.2)	268,514 (44.7)	
Household income, *n* (%)				0.038
High (70−100th percentile)	684,264 (42.5)	261,606 (43.5)	250,626 (41.7)	
Middle (30−69th percentile)	660,916 (41.1)	243,423 (40.5)	249,392 (41.5)	
Low (0−29th percentile)	263,360 (16.4)	95,959 (16.0)	100,970 (16.8)	
Preterm birth, ≤36 week, *n* (%)	33,294 (2.1)	7,407 (1.2)	7,417 (1.2)	<0.001
Low birth weight, ≤2499 g, *n* (%)	26,001 (1.6)	5,451 (0.9)	5,423 (0.9)	<0.001

SMD standardized mean differences.

[a]An SMD of <0.1 indicates no major imbalance. All SMD values were <0.07 in each propensity-score-matched cohort.

Supplementary Table 4 | Statistical model to determine the relationship between types of infant feeding and overall hospital admissions (1:1 propensity-score-matched cohort; *n* = 1,201,976)

Parameter	Individuals with at least one event	Admission events	Person-years	Incidence rate of admission events[a]	Incidence rate ratio of admission events (95% CI)	
					Minimally-adjusted[b]	Adjusted[c]
Feeding types (overall)						
Fully formula feeding	406,107	1,175,203	4,769,116.5	24.64	1.0 (reference)	1.0 (reference)
Exclusive breastfeeding	379,525	994,951	4,883,566.6	20.37	**0.85 (0.84 to 0.85)**	**0.85 (0.85 to 0.85)**
Male						
Fully formula feeding	213,530	631,121	2,436,212.8	25.91	1.0 (reference)	1.0 (reference)
Exclusive breastfeeding	187,157	503,197	2,316,294.5	21.72	**0.86 (0.85 to 0.86)**	**0.85 (0.85 to 0.86)**
Female						
Fully formula feeding	192,577	544,082	2,332,903.7	23.32	1.0 (reference)	1.0 (reference)
Exclusive breastfeeding	192,368	491,754	2,567,272.1	19.15	**0.85 (0.84 to 0.85)**	**0.85 (0.85 to 0.86)**
Calendar period of birth (2008-2010)						
Fully formula feeding	144,105	426,336	2,099,897.5	20.30	1.0 (reference)	1.0 (reference)
Exclusive breastfeeding	151,271	401,759	2,356,973.8	17.05	**0.84 (0.84 to 0.85)**	**0.85 (0.85 to 0.86)**
Calendar period of birth (2011-2012)						
Fully formula feeding	126,302	371,959	1,475,907.4	25.20	1.0 (reference)	1.0 (reference)
Exclusive breastfeeding	112,614	299,405	1,416,770.8	21.13	**0.84 (0.84 to 0.85)**	**0.85 (0.84 to 0.85)**
Calendar period of birth (2013-2015)						
Fully formula feeding	135,700	376,908	1,193,311.6	31.59	1.0 (reference)	1.0 (reference)
Exclusive breastfeeding	115,640	293,787	1,109,822.0	26.47	**0.85 (0.85 to 0.86)**	**0.85 (0.85 to 0.86)**
Rural						
Fully formula feeding	225,655	664,202	2,630,075.0	25.25	1.0 (reference)	1.0 (reference)
Exclusive breastfeeding	216,393	574,382	2,763,128.1	20.79	**0.84 (0.84 to 0.85)**	**0.85 (0.84 to 0.85)**
Urban						
Fully formula feeding	180,452	511,001	2,139,041.5	23.89	1.0 (reference)	1.0 (reference)
Exclusive breastfeeding	163,132	420,569	2,120,438.5	19.83	**0.85 (0.85 to 0.86)**	**0.86 (0.85 to 0.86)**

CI confidence interval.

[a]Hospital admission rate is expressed as per 100 person-years.

bAdjusted model: adjusted for infant sex and calendar period of birth (2008-2010, 2011-2012, and 2013-2015).

[c]Adjusted model: adjusted for infant sex, calendar period of birth (2008-2010, 2011-2012, and 2013-2015), birth season (spring, summer, autumn, and winter), region of residence (rural and urban), household income (high, middle, and low), preterm birth, and low birth weight.

We performed the negative binomial regression model (endpoint, incidence rate of any hospital admission) with incidence rate ratios and 95% CIs.

Numbers in bold indicate significant differences (two sided $p < 0.05$).

Supplementary Table 5 | Hospital admissions during childhood by types of infant feeding, stratified by age at admission (1:1 propensity-score-matched cohort; *n* = 1,201,976)

Parameter	Individuals with at least one event	Admission events	Person-years	Incidence rate of admission events[a]	IRR of admission events (95% CI)	
					Sex-adjusted	Adjusted[b]
<1 year						
Fully formula feeding	251,133	365,883	600,958.0	60.88	1.0 (reference)	1.0 (reference)
Exclusive breastfeeding	224,725	307,949	600,959.1	51.24	**0.84 (0.84 to 0.85)**	**0.85 (0.85 to 0.86)**
1-2 years						
Fully formula feeding	209,925	411,110	1,201,662.6	34.21	1.0 (reference)	1.0 (reference)
Exclusive breastfeeding	184,351	337,506	1,201,660.9	28.09	**0.82 (0.82 to 0.83)**	**0.83 (0.83 to 0.84)**
3-4 years						
Fully formula feeding	127,065	208,735	1,200,571.0	17.39	1.0 (reference)	1.0 (reference)
Exclusive breastfeeding	114,733	179,538	1,200,833.7	14.95	**0.86 (0.85 to 0.87)**	**0.87 (0.86 to 0.88)**
5-6 years						
Fully formula feeding	83,846	120,722	982,695.3	12.28	1.0 (reference)	1.0 (reference)
Exclusive breastfeeding	76,689	106,586	1,021,558.4	10.43	**0.88 (0.87 to 0.89)**	**0.88 (0.87 to 0.89)**
7-10 years						
Fully formula feeding	46,608	68,753	783,229.7	8.78	1.0 (reference)	1.0 (reference)
Exclusive breastfeeding	44,597	63,372	858,554.6	7.38	**0.92 (0.91 to 0.94)**	**0.86 (0.85 to 0.88)**

IRR incidence rate ratio, *CI* confidence interval.

[a]Hospital admission rate is expressed as per 100 person-years.

[b]Adjusted model: adjusted for infant sex, calendar period of birth (2008-2010, 2011-2012, and 2013-2015), birth season (spring, summer, autumn, and winter), region of residence (rural and urban), household income (high, middle, and low), preterm birth, and low birth weight.

We performed the negative binomial regression model (endpoint, incidence rate of any hospital admission) with incidence rate ratios and 95% CIs.

Numbers in bold indicate significant differences (two sided $p < 0.05$).

Supplementary Table 6 | Relationship of types of infant feeding with subsequent hospital admissions, stratified by cause of morbidity in the 1:1 propensity-score-matched cohort (*n* = 1,201,976)

Parameter	IRR of admission events (95% CI)	
	**Sex-adjusted**	**Adjusted[a]**
Infection		
Fully formula feeding	1.0 (reference)	1.0 (reference)
Exclusive breastfeeding	**0.83 (0.82 to 0.83)**	**0.83 (0.83 to 0.84)**
Respiratory		
Fully formula feeding	1.0 (reference)	1.0 (reference)
Exclusive breastfeeding	**0.94 (0.92 to 0.96)**	**0.93 (0.91 to 0.95)**
Gastrointestinal tract		
Fully formula feeding	1.0 (reference)	1.0 (reference)
Exclusive breastfeeding	**0.84 (0.83 to 0.84)**	**0.84 (0.83 to 0.84)**
Oral cavity		
Fully formula feeding	1.0 (reference)	1.0 (reference)
Exclusive breastfeeding	**0.85 (0.78 to 0.93)**	**0.84 (0.77 to 0.92)**
Mental health		
Fully formula feeding	1.0 (reference)	1.0 (reference)
Exclusive breastfeeding	1.09 (0.84 to 1.43)	1.11 (0.85 to 1.45)
Injury/External		
Fully formula feeding	1.0 (reference)	1.0 (reference)
Exclusive breastfeeding	1.01 (0.99 to 1.03)	1.00 (0.98 to 1.02)
Genitourinary tract		
Fully formula feeding	1.0 (reference)	1.0 (reference)
Exclusive breastfeeding	**0.78 (0.75 to 0.82)**	**0.80 (0.76 to 0.84)**

IRR incidence rate ratio, *CI* confidence interval.

[a]Adjusted model: adjusted for infant sex, calendar period of birth (2008-2010, 2011-2012, and 2013-2015), birth season (spring, summer, autumn, and winter), region of residence (rural and urban), household income (high, middle, and low), preterm birth, and low birth weight.

We performed the negative binomial regression model (endpoint, incidence rate of any hospital admission) with incidence rate ratios and 95% CIs.

Numbers in bold indicate significant differences (two sided $p < 0.05$).

Supplementary Table 7 | Hospital admissions during childhood by types of infant feeding, stratified by age at admission and cause of morbidity in the 1:1 propensity-score-matched cohort (*n* = 1,201,976)

	**Age at admission, years**				
	**<1 years**	**1-2 years**	**3-4 years**	**5-6 years**	**7-10 years**
Infection					
Sex-adjusted IRR (95% CI)	**0.787 (0.781 to 0.794)**	**0.815 (0.809 to 0.821)**	**0.853 (0.845 to 0.862)**	**0.862 (0.851 to 0.873)**	**0.898 (0.882 to 0.914)**
Adjusted IRR[a] (95% CI)	**0.794 (0.787 to 0.801)**	**0.827 (0.821 to 0.833)**	**0.862 (0.853 to 0.871)**	**0.857 (0.846 to 0.868)**	**0.843 (0.828 to 0.858)**
Respiratory					
Sex-adjusted IRR (95% CI)	**0.614 (0.542 to 0.696)**	**0.768 (0.726 to 0.811)**	**0.912 (0.879 to 0.946)**	0.999 (0.966 to 1.033)	1.065 (1.014 to 1.119)
Adjusted IRR[a] (95% CI)	**0.629 (0.555 to 0.712)**	**0.783 (0.738 to 0.830)**	**0.925 (0.890 to 0.960)**	**0.965 (0.965 to 0.965)**	1.007 (0.957 to 1.059)
Gastrointestinal tract					
Sex-adjusted IRR (95% CI)	**0.766 (0.753 to 0.780)**	**0.829 (0.818 to 0.841)**	**0.860 (0.846 to 0.875)**	**0.878 (0.859 to 0.898)**	**0.914 (0.888 to 0.940)**
Adjusted IRR[a] (95% CI)	**0.773 (0.760 to 0.787)**	**0.834 (0.823 to 0.845)**	**0.867 (0.853 to 0.883)**	**0.869 (0.850 to 0.889)**	**0.850 (0.827 to 0.875)**
Oral cavity					
Sex-adjusted IRR (95% CI)	**0.694 (0.544 to 0.886)**	**0.827 (0.708 to 0.966)**	**0.771 (0.628 to 0.947)**	0.971 (0.800 to 1.179)	1.008 (0.784 to 1.295)
Adjusted IRR[a] (95% CI)	**0.694 (0.542 to 0.890)**	**0.823 (0.703 to 0.965)**	**0.769 (0.624 to 0.948)**	0.972 (0.797 to 1.186)	0.951 (0.740 to 1.222)
Mental health					
Sex-adjusted IRR (95% CI)	2.000 (0.684 to 5.851)	0.727 (0.430 to 1.230)	0.833 (0.464 to 1.496)	1.526 (0.910 to 2.560)	1.069 (0.645 to 1.773)
Adjusted IRR[a] (95% CI)	1.988 (0.678 to 5.826)	0.748 (0.441 to 1.266)	**0.858 (0.858 to 0.858)**	1.265 (0.747 to 2.143)	1.003 (0.493 to 2.040)
Injury/External					
Sex-adjusted IRR (95% CI)	**0.956 (0.919 to 0.995)**	1.008 (0.969 to 1.048)	1.014 (0.972 to 1.057)	1.030 (0.989 to 1.074)	1.040 (0.999 to 1.082)
Adjusted IRR[a] (95% CI)	0.972 (0.932 to 1.013)	1.020 (0.978 to 1.065)	1.019 (0.974 to 1.067)	1.023 (0.978 to 1.071)	0.979 (0.935 to 1.024)
Genitourinary tract					
Sex-adjusted IRR (95% CI)	**0.737 (0.687 to 0.792)**	**0.788 (0.714 to 0.870)**	**0.728 (0.646 to 0.822)**	0.910 (0.787 to 1.051)	0.927 (0.782 to 1.099)
Adjusted IRR[a] (95% CI)	**0.770 (0.715 to 0.829)**	**0.814 (0.737 to 0.899)**	**0.739 (0.655 to 0.834)**	0.910 (0.787 to 1.052)	0.866 (0.730 to 1.027)

IRR incidence rate ratio, *CI* confidence interval.

[a]Adjusted model: adjusted for infant sex, calendar period of birth (2008-2010, 2011-2012, and 2013-2015), birth season (spring, summer, autumn, and winter), region of residence (rural and urban), household income (high, middle, and low), preterm birth, and low birth weight.

We performed the negative binomial regression model (endpoint, incidence rate of any hospital admission) with incidence rate ratios and 95% CIs.

Numbers in bold indicate significant differences (two sided $p < 0.05$).

Supplementary Table 8 | ICD-10 code tables

Covariates	ICD-10 code
Infection	A02.1 Salmonella sepsis<br>A17.0 Tuberculous meningitis<br>A17.1 Meningeal tuberculoma<br>A17.8 Other tuberculosis of nervous system<br>A17.9 Tuberculosis of nervous system, unspecified<br>A18.0 Tuberculosis of bones and joints<br>A19.0 Acute miliary tuberculosis of a single specified site<br>A19.1 Acute miliary tuberculosis of multiple sites<br>A20.3 Plague meningitis<br>A20.7 Septicemic plague<br>A21.7 Generalized tularaemia<br>A22.7 Anthrax sepsis<br>A23.0 Brucellosis due to Brucella melitensis<br>A23.1 Brucellosis due to Brucella abortus<br>A23.2 Brucellosis due to Brucella suis<br>A23.3 Brucellosis due to Brucella canis<br>A23.8 Other brucellosis<br>A23.9 Brucellosis, unspecified<br>A24.1 Acute and fulminating melioidosis<br>A25.0 Spirillosis<br>A25.1 Streptobacillosis<br>A25.9 Rat-bite fever, unspecified<br>A32.1 Listerial meningitis and meningoencephalitis<br>A32.7 Listerial sepsis<br>A39.0 Meningococcal meningitis<br>A39.1 Waterhouse-Friderichsen syndrome<br>A39.2 Acute meningococcaemia<br>A39.3 Chronic meningococcaemia<br>A39.4 Meningococcemia, unspecified<br>A39.5 Meningococcal heart disease<br>A39.8 Other meningococcal infections<br>A39.8Other meningococcal infections<br>A39.9 Meningococcal infection, unspecified<br>A40.0 Sepsis due to streptococcus, group A<br>A40.1 Sepsis due to streptococcus, group B<br>A40.2 Sepsis due to streptococcus, group D<br>A40.3 Sepsis due to Streptococcus pneumoniae<br>A40.8 Other streptococcal sepsis<br>A40.9 Streptococcal sepsis, unspecified<br>A41.0 Sepsis due to Staphylococcus aureus<br>A41.1 Sepsis due to other specified staphylococcus<br>A41.2 Sepsis due to unspecified staphylococcus<br>A41.3 Sepsis due to Hemophilus influenzae<br>A41.4 Sepsis due to anaerobes<br>A41.51 Sepsis due to Escherichia coli [E. Coli]<br>A41.52 Sepsis due to Pseudomonas<br>A41.58 Sepsis due to other Gram-negative organisms<br>A41.8 Other specified septicaemia<br>A41.9 Sepsis, unspecified<br>A44.0 Systemic bartonellosis<br>A48.3 Toxic shock syndrome<br>A52.0 Cardiovascular syphilis<br>A52.1 Symptomatic neurosyphilis<br>A52.1 (no description found)<br>A52.2 Asymptomatic neurosyphilis<br>A52.3 Neurosyphilis, unspecified<br>A52.7 Other symptomatic late syphilis<br>A52.7 (no description found)<br>A52.8 Late syphilis, latent<br>A65 Nonvenereal syphilis<br>A74.0 Chlamydial conjunctivitis<br>A74.8 Other chlamydial diseases<br>A78 Q fever<br>A79.0 Trench fever<br>A79.1 Rickettsialpox due to Rickettsia akari<br>A79.8 Other specified rickettsioses<br>A79.9 Rickettsiosis, unspecified<br>B95.1 Streptococcus, group B, as the cause of diseases classified elsewhere<br>G00.0 Haemophilus meningitis

G00.1 Pneumococcal meningitis
G00.1 (no description found)
G00.2 Streptococcal meningitis
G00.3 Staphylococcal meningitis
G00.8 Other bacterial meningitis
G00.9 Bacterial meningitis, unspecified
G01 Meningitis in bacterial diseases classified elsewhere
G03.1 Chronic meningitis
G05.0 Encephalitis, myelitis and encephalomyelitis in bacterial diseases classified elsewhere
G06.0 Intracranial abscess and granuloma
G06.1 Intraspinal abscess and granuloma
G06.2 Extradural and subdural abscess, unspecified
G07 Intracranial and intraspinal abscess and granuloma in disease classified elsewhere
I30.1 Infective pericarditis
I32.0 Pericarditis in bacterial diseases classified elsewhere
I33.0 Acute and subacute infective endocarditis
I41.0 Myocarditis in bacterial diseases classified elsewhere
J39.0 Retropharyngeal and parapharyngeal abscess
J39.1 Other abscess of pharynx
J85.3 Abscess of mediastinum
M00.90 Pyogenic arthritis, unspecified, multiple sites
M00.91 Pyogenic arthritis, unspecified, shoulder region
M00.92 Pyogenic arthritis, unspecified, upper arm
M00.93 Pyogenic arthritis, unspecified, forearm
M00.94 Pyogenic arthritis, unspecified, hand
M00.95 Pyogenic arthritis, unspecified, pelvic region and thigh
M00.96 Pyogenic arthritis, unspecified, lower leg
M00.97 Pyogenic arthritis, unspecified, ankle and foot
M00.98 Pyogenic arthritis, unspecified, other site
M00.99 Pyogenic arthritis, unspecified, site unspecified
M01.0 Meningococcal arthritis
M01.1 Tuberculous arthritis
M46.2 Osteomyelitis of vertebra
M46.3 Infection of intervertebral disc (pyogenic)
M46.4 Discitis, unspecified
M46.5 Other infective spondylopathies
M49.0 Tuberculosis of spine
M49.1 Brucella spondylitis
M49.2 Enterobacterial spondylitis
M49.3 Spondylopathy in other infectious and parasitic diseases classified elsewhere
M72.6 Necrotizing fasciitis
M73.0 Gonococcal bursitis
M73.1 Syphilitic bursitis
M86.0 Acute haematogenous osteomyelitis
M86.10 Other acute osteomyelitis, unspecified site
M86.11 Other acute osteomyelitis, shoulder region
M86.12 Other acute osteomyelitis, upper arm
M86.13 Other acute osteomyelitis, forearm
M86.14 Other acute osteomyelitis, hand
M86.15 Other acute osteomyelitis, pelvic region and thigh
M86.16 Other acute osteomyelitis, lower leg
M86.17 Other acute osteomyelitis, ankle and foot
M86.18 Other acute osteomyelitis, other site
M86.19 Other acute osteomyelitis, multiple sites
M86.2 Subacute osteomyelitis
M86.4 Chronic osteomyelitis with draining sinus
M86.5 Other chronic osteomyelitis
M86.60 Other chronic osteomyelitis, unspecified site
M86.67 Other chronic osteomyelitis, ankle and foot
M86.68 Other chronic osteomyelitis, other site
M86.69 Other chronic osteomyelitis, multiple sites
M86.8 Other osteomyelitis
M86.90 Unspecified osteomyelitis, multiple sites
M86.91 Unspecified osteomyelitis, shoulder region
M86.92 Unspecified osteomyelitis, upper arm
M86.93 Unspecified osteomyelitis, forearm
M86.94 Unspecified osteomyelitis, hand
M86.95 Unspecified osteomyelitis, pelvic region and thigh
M86.96 Unspecified osteomyelitis, lower leg
M86.97 Unspecified osteomyelitis, ankle and foot
M86.98 Unspecified osteomyelitis, other site
M86.99 Unspecified osteomyelitis, site unspecified
M90.0 Tuberculosis of bone

P36.0 Sepsis of newborn due to streptococcus, group B
P36.1 Sepsis of newborn due to other and unspecified streptococci
P36.2 Sepsis of newborn due to Staphylococcus aureus
P36.3 Sepsis of newborn due to other and unspecified staphylococci
P36.4 Sepsis of newborn due to Escherichia coli
P36.5 Sepsis of newborn due to anaerobes
P36.8 Other bacterial sepsis of newborn
P36.9 Bacterial sepsis of newborn, unspecified
P37.0 Congenital tuberculosis
P37.2 Neonatal (disseminated) listeriosis
A00.0 Cholera due to Vibrio cholerae 01, biovar cholerae
A00.1 Cholera due to Vibrio cholerae 01, biovar eltor
A00.9 Cholera, unspecified
A01.0 Typhoid fever
A01.1 Paratyphoid fever A
A01.2 Paratyphoid fever B
A01.3 Paratyphoid fever C
A01.4 Paratyphoid fever, unspecified
A02.0 Salmonella enteritis
A02.2 Localised salmonella infections
A02.8 Other specified salmonella infections
A02.9 Salmonella infection, unspecified
A03.0 Shigellosis due to Shigella dysenteriae
A03.1 Shigellosis due to Shigella flexneri
A03.2 Shigellosis due to Shigella boydii
A03.3 Shigellosis due to Shigella sonnei
A03.8 Other shigellosis
A03.9 Shigellosis, unspecified
A04.0 Enteropathogenic Escherichia coli infection
A04.1 Enterotoxigenic Escherichia coli infection
A04.2 Enteroinvasive Escherichia coli infection
A04.3 Enterohemorrhagic Escherichia coli infection
A04.4 Other intestinal Escherichia coli infections
A04.5 Campylobacter enteritis
A04.6 Enteritis due to Yersinia enterocolitica
A04.7 Enterocolitis due to Clostridium difficile
A04.8 Other specified bacterial intestinal infections
A04.9 Bacterial intestinal infection, unspecified
A05.0 Foodborne staphylococcal intoxication
A05.1 Botulism food poisoning
A05.2 Foodborne Clostridium perfringens [Clostridium welchii] intoxication
A05.3 Foodborne Vibrio parahaemolyticus intoxication
A05.4 Foodborne Bacillus cereus intoxication
A05.8 Other specified bacterial foodborne intoxications
A05.9 Bacterial foodborne intoxication, unspecified
A06.0 Acute amebic dysentery
A06.1 Chronic intestinal amebiasis
A06.2 Amebic nondysenteric colitis
A06.3 Amoeboma of intestine
A06.4 Amebic liver abscess
A06.9 Amebiasis, unspecified
A07.0 Balantidiasis
A07.1 Giardiasis [lambliasis]
A07.2 Cryptosporidiosis
A07.3 Isosporiasis
A07.8 Other specified protozoal intestinal diseases
A07.9 Protozoal intestinal disease, unspecified
A08.0 Rotaviral enteritis
A08.1 Acute gastroenteropathy due to Norwalk agent
A08.2 Adenoviral enteritis
A08.3 Other viral enteritis
A08.4 Viral intestinal infection, unspecified
A08.5 Other specified intestinal infections
A09 Infectious gastroenteritis and colitis, unspecified
A09.0 Other and unspecified gastroenteritis and colitis of infectious origin
A09.9 Gastroenteritis and colitis of unspecified origin
A21.3 Gastrointestinal tularemia
A22.2 Gastrointestinal anthrax
B37.88 Candidiasis of other sites
B71.0 Hymenolepiasis
B71.9 Cestode infection, unspecified
B76.0 Ancylostomiasis
B78.9 Strongyloidiasis, unspecified

B81.0 Anisakiasis
B96.81 Helicobacter pylori [H. pylori] as the cause of diseases classified to other chapters
K52.8 Other specified noninfective gastroenteritis and colitis
A06.5 Amebic lung abscess
A15.0 Tuberculosis of lung
A15.1 Tuberculosis of lung, confirmed by culture only
A15.2 Tuberculosis of lung, confirmed histologically
A15.3 Tuberculosis of lung, confirmed by unspecified means
A15.4 Tuberculosis of intrathoracic lymph nodes
A15.5 Tuberculosis of larynx, trachea and bronchus
A15.6 Tuberculosis pleurisy
A15.7 Primary respiratory tuberculosis
A15.8 Other respiratory tuberculosis
A15.9 Respiratory tuberculosis unspecified, confirmed bacteriologically and histologically
A16.0 Tuberculosis of lung, bacteriologically and histologically negative
A16.1 Tuberculosis of lung, bacteriological and histological examination not done
A16.2 Tuberculosis of lung, without mention of bacteriological or histological confirmation
A16.3 Tuberculosis of intrathoracic lymph nodes, without mention of bacteriological or histological confirmation
A16.4 Tuberculosis of larynx, trachea and bronchus, without mention of bacteriological or histological confirmation
A16.5 Tuberculous pleurisy, without mention of bacteriological or histological confirmation
A16.7 Primary respiratory tuberculosis, without mention of bacteriological or histological confirmation
A16.8 Other respiratory tuberculosis, without mention of bacteriological or histological confirmation
A16.9 Respiratory tuberculosis unspecified, without mention of bacteriological or histological confirmation
A19.2 Acute miliary tuberculosis, unspecified
A19.8 Other miliary tuberculosis
A19.9 Miliary tuberculosis, unspecified
A20.2 Pneumonic plague
A21.2 Pulmonary tularemia
A22.1 Pulmonary anthrax
A24.0 Glanders
A31.0 Pulmonary mycobacterial infection
A37.0 Whooping cough due to Bordetella pertussis
A37.1 Whooping cough due to Bordetella parapertussis
A37.8 Whooping cough due to other Bordetella species
A37.9 Whooping cough, unspecified
A42.0 Pulmonary actinomycosis
A48.1 Legionnaires disease
A70 Chlamydia psittaci infections
A70 (no description found)
B01.2 Varicella pneumonia
B05.2 Measles complicated by pneumonia
B34.0 Adenovirus infection, unspecified
B37.1 Pulmonary candidiasis
B38.0 Acute pulmonary coccidioidomycosis
B38.1 Chronic pulmonary coccidioidomycosis
B38.2 Pulmonary coccidioidomycosis, unspecified
B39.0 Acute pulmonary histoplasmosis capsulati
B39.1 Chronic pulmonary histoplasmosis capsulati
B39.2 Pulmonary histoplasmosis capsulati, unspecified
B39.3 Disseminated histoplasmosis capsulati
B39.4 Histoplasmosis capsulati, unspecified
B39.5 Histoplasmosis duboisii
B39.9 Histoplasmosis, unspecified
B40.0 Acute pulmonary blastomycosis
B40.1 Chronic pulmonary blastomycosis
B40.2 Pulmonary blastomycosis, unspecified
B40.3 Cutaneous blastomycosis
B40.7 Disseminated blastomycosis
B40.8 Other forms of blastomycosis
B41.0 Pulmonary paracoccidioidomycosis
B42.0 Pulmonary sporotrichosis
B44.0 Invasive pulmonary aspergillosis
B44.1 Other pulmonary aspergillosis
B45.0 Pulmonary cryptococcosis
B58.3 Pulmonary toxoplasmosis
B59 Pneumocystosis
J05.0 Acute obstructive laryngitis [croup]
J09 Influenza due to certain identified influenza virus
J10.0 Influenza with pneumonia, influenza virus identified
J10.1 Influenza due to other influenza virus with respiratory manifestations
J11.0 Influenza with pneumonia, virus not identified
J11.1 Influenza with other respiratory manifestations, virus not identified

J12.0 Adenoviral pneumonia
J12.1 Respiratory syncytial virus pneumonia
J12.2 Parainfluenza virus pneumonia
J12.3 Human metapneumovirus pneumonia
J12.8 Other viral pneumonia
J12.9 Viral pneumonia, unspecified
J13 Pneumonia due to Streptococcus pneumoniae
J14 Pneumonia due to Hemophilus influenzae
J15.0 Pneumonia due to Klebsiella pneumoniae
J15.1 Pneumonia due to Pseudomonas
J15.2 Pneumonia due to staphylococcus
J15.3 Pneumonia due to streptococcus, group B
J15.4 Pneumonia due to other streptococci
J15.5 Pneumonia due to Escherichia coli
J15.6 Pneumonia due to other aerobic Gram-negative bacteria
J15.7 Pneumonia due to Mycoplasma pneumoniae
J15.8 Pneumonia due to other specified bacteria
J15.9 Unspecified bacterial pneumonia
J16.0 Chlamydial pneumonia
J16.8 Pneumonia due to other specified infectious organisms
J17.0 Pneumonia in bacterial diseases classified elsewhere
J17.1 Pneumonia in viral diseases classified elsewhere
J17.2 Pneumonia in mycoses
J17.3 Pneumonia in parasitic diseases
J17.8 Pneumonia in other diseases classified elsewhere
J18.0 Bronchopneumonia, unspecified organism
J18.1 Lobar pneumonia, unspecified
J18.8 Other pneumonia, unspecified organism
J18.9 Pneumonia, unspecified
J20.0 Acute bronchitis due to Mycoplasma pneumoniae
J20.1 Acute bronchitis due to Hemophilus influenza
J20.2 Acute bronchitis due to streptococcus
J20.3 Acute bronchitis due to coxsackievirus
J20.4 Acute bronchitis due to parainfluenza virus
J20.5 Acute bronchitis due to respiratory syncytial virus
J20.6 Acute bronchitis due to rhinovirus
J20.7 Acute bronchitis due to echovirus
J20.8 Acute bronchitis due to other specified organisms
J20.9 Acute bronchitis, unspecified
J21.0 Acute bronchiolitis due to respiratory syncytial virus
J21.1 Acute bronchiolitis due to human megapneumovirus
J21.8 Acute bronchiolitis due to other specified organisms
J21.9 Acute bronchiolitis, unspecified
J22 Unspecified acute lower respiratory infection
J22.0 (no description found)
J40 Bronchitis, not specified as acute or chronic
J41.0 Simple chronic bronchitis
J41.1 Mucopurulent chronic bronchitis
J41.8 Mixed simple and mucopurulent chronic bronchitis
J42 Unspecified chronic bronchitis
J44.0 Chronic obstructive pulmonary disease with acute lower respiratory infection
J47 Bronchiectasis
J65 Pneumoconiosis associated with tuberculosis
J85.0 Gangrene and necrosis of lung
J85.1 Abscess of lung with pneumonia
J85.2 Abscess of lung without pneumonia
J86.0 Pyothorax with fistula
J86.9 Pyothorax without fistula
P23.0 Congenital pneumonia due to viral agent
P23.2 Congenital pneumonia due to staphylococcus
P23.3 Congenital pneumonia due to staphylococcus, group B
P23.4 Congenital pneumonia due to Escherichia coli
P23.5 Congenital pneumonia due to Pseudomonas
P23.6 Congenital pneumonia due to other bacterial agents
P23.8 Congenital pneumonia due to other organisms
P23.9 Congenital pneumonia, unspecified
A31.1 Cutaneous mycobacterial infection
A46 Erysipelas
B00.0 Eczema herpeticum
B35.0 Tinea barbae and tinea capitis
B35.1 Tinea unguium
B35.2 Tinea manuum
B35.3 Tinea pedis

B35.4 Tinea corporis
B35.5 Tinea imbricate
B35.6 Tinea cruris
B35.8 Other dermatophytoses
B35.9 Dermatophytosis, unspecified
B36.0 Pityriasis versicolour
B36.1 Tinea nigra
B36.2 White piedra
B36.3 Black piedra
B36.8 Other specified superficial mycoses
B37.2 Candidiasis of skin and nail
B85.0 Pediculosis due to Pediculus humanus capitis
B85.1 Pediculosis due to Pediculus humanus corporis
B85.2 Pediculosis, unspecified
B85.3 Phthiriasis
B85.4 Mixed pediculosis and phthiriasis
B86 Scabies
B87.9 Myiasis, unspecified
B88.0 Other acariasis
B88.1 Tungiasis [sandflea infestation]
B88.2 Other arthropod infestations
B88.3 External hirudiniasis
B88.8 Other specified infestations
B88.9 Infestation, unspecified
H60.3 Other infective otitis externa
L00 Staphylococcal scalded skin syndrome
L01.0 Impetigo [any organism] [any site]
L02.0 Cutaneous abscess, furuncle and carbuncle of face
L02.1 Cutaneous abscess, furuncle and carbuncle of neck
L02.2 Cutaneous abscess, furuncle and carbuncle of trunk
L02.3 Cutaneous abscess, furuncle and carbuncle of buttock
L02.4 Cutaneous abscess, furuncle and carbuncle of limb
L02.8 Cutaneous abscess, furuncle and carbuncle of other sites
L02.9 Cutaneous abscess, furuncle and carbuncle, unspecified
L03.01 Cellulitis of finger
L03.02 Cellulitis of toe
L03.10 Cellulitis of upper limb
L03.11 Cellulitis of lower limb
L03.2 Cellulitis of face
L03.3 Cellulitis of trunk
L03.8 Cellulitis of other sites
L03.9 Cellulitis, unspecified
L05.0 Pilonidal cyst with abscess
L05.9 Pilonidal cyst without abscess
L08.0 Pyoderma
L08.1 Erythrasma
L08.8 Other specified local infections of skin and subcutaneous tissue
L08.9 Local infection of the skin and subcutaneous tissue, unspecified
L13.0 Dermatitis herpetiformis
L30.3 Infective dermatitis
L88 Pyoderma gangrenosum
M60.09 Infective myositis, multiple sites
M63.0 Myositis in bacterial diseases classified elsewhere
M63.1 Myositis in protozoal and parasitic infections classified elsewhere
M63.2 Myositis in other infectious diseases classified elsewhere
M65.0 Abscess of tendon sheath
M65.1 Other infective (teno)synovitis
M68.0 Synovitis and tenosynovitis in bacterial diseases classified elsewhere
M71.0 Abscess of bursa
M71.1 Other infective bursitis
O91.00 Infection of nipple associated with childbirth, without mention of attachment difficulty
O91.10 Abscess of breast associated with childbirth, without mention of attachment difficulty
P39.0 Neonatal infective mastitis
P39.4 Neonatal skin infection
A36.0 Pharyngeal diphtheria
A36.1 Nasopharyngeal diphtheria
A36.2 Laryngeal diphtheria
B05.3 Measles complicated by otitis media
H65.0 Acute serous otitis media
H65.1 Other acute nonsuppurative otitis media
H65.2 Chronic serous otitis media
H65.3 Chronic mucoid otitis media
H65.4 Other chronic nonsuppurative otitis media

H65.9 Nonsuppurative otitis media, unspecified
H66.0 Acute suppurative otitis media
H66.1 Chronic tubotympanic suppurative otitis media
H66.2 Chronic atticoantral suppurative otitis media
H66.3 Other chronic suppurative otitis media
H66.4 Suppurative otitis media, unspecified
H66.9 Otitis media, unspecified
H67.0 Otitis media in bacterial diseases classified elsewhere
H67.8 Otitis media in other diseases classified elsewhere
H68.0 Eustachian salpingitis
H70.0 Acute mastoiditis
H70.1 Chronic mastoiditis
H70.2 Petrositis
H70.8 Other mastoiditis and related conditions
H70.9 Mastoiditis, unspecified
H72.0 Central perforation of tympanic membrane
H72.1 Attic perforation of tympanic membrane
H72.2 Other marginal perforations of tympanic membrane
H72.8 Other perforations of tympanic membrane
H72.9 Perforation of tympanic membrane, unspecified
H73.0 Acute myringitis
H75.0 Mastoiditis in infectious and parasitic diseases classified elsewhere
H83.0 Labyrinthitis
H92.1 Otorrhoea
J00 Acute nasopharyngitis [common cold]
J01.0 Acute maxillary sinusitis
J01.1 Acute frontal sinusitis
J01.2 Acute ethmoidal sinusitis
J01.3 Acute sphenoidal sinusitis
J01.4 Acute pansinusitis
J01.8 Other acute sinusitis
J01.9 Acute sinusitis, unspecified
J02.0 Streptococcal pharyngitis
J02.8 Acute pharyngitis due to other specified organisms
J02.9 Acute pharyngitis, unspecified
J03.0 Streptococcal tonsillitis
J03.8 Acute tonsillitis due to other specified organisms
J03.9 Acute tonsillitis, unspecified
J04.0 Acute laryngitis
J04.1 Acute tracheitis
J04.2 Acute laryngotracheitis
J05.1 Acute epiglottitis
J06.0 Acute laryngopharyngitis
J06.8 Other acute upper respiratory infections of multiple sites
J06.9 Acute upper respiratory infection, unspecified
J32.0 Chronic maxillary sinusitis
J32.1 Chronic frontal sinusitis
J32.2 Chronic ethmoidal sinusitis
J32.3 Chronic sphenoidal sinusitis
J32.4 Chronic pansinusitis
J32.8 Other chronic sinusitis
J32.9 Chronic sinusitis, unspecified
J34.0 Abscess, furuncle and carbuncle of nose
J35.0 Chronic tonsillitis
J36 Peritonsillar abscess
A51.0 Primary genital syphilis
A51.1 Primary anal syphilis
A51.2 Primary syphilis of other sites
A51.3 Secondary syphilis of skin and mucous membranes
A51.4 Other secondary syphilis
A51.5 Early syphilis, latent
A51.9 Early syphilis, unspecified
A52.9 Late syphilis, unspecified
A53.0 Latent syphilis, unspecified as early or late
A53.9 Syphilis, unspecified
A54.0 Gonococcal infection of lower genitourinary tract without periurethral or accessory gland abscess
A54.1 Gonococcal infection of lower genitourinary tract with periurethral and accessory gland abscess
A54.2 Gonococcal pelviperitonitis and other gonococcal genitourinary infections
A54.3 Gonococcal infection of eye
A54.3 (no description found)
A54.4 Gonococcal infection of musculoskeletal system
A54.5 Gonococcal pharyngitis
A54.6 Gonococcal infection of anus and rectum

A54.8 Other gonococcal infections
A54.8 (no description found)
A54.9 Gonococcal infection, unspecified
A55 Chlamydial lymphogranuloma (venereum)
A56.0 Chlamydial infection of lower genitourinary tract
A56.1 Chlamydial infection of pelviperitoneum and other genitourinary organs
A56.2 Chlamydial infection of genitourinary tract, unspecified
A56.3 Chlamydial infection of anus and rectum
A56.4 Chlamydial infection of pharynx
A56.8 Sexually transmitted chlamydial infection of other sites
A57 Chancroid
A58 Granuloma inguinale
A59.0 Urogenital trichomoniasis
A59.0 (no description found)
A60.0 Herpesviral infection of genitalia and urogenital tract
A60.1 Herpesviral infection of perianal skin and rectum
A60.9 Anogenital herpesviral infection, unpsecified
A63.0 Anogenital (venereal) warts
A63.8 Other specified predominantly sexually transmitted diseases
A64 Unspecified sexually transmitted disease
B37.3 Candidiasis of vulva and vagina
B37.4 Candidiasis of other urogenital sites
N13.6 Pyonephrosis
N15.1 Renal and perinephric abscess
N30.0 Acute cystitis
N30.8 Other cystitis, abscess of bladder
N34.0 Urethral abscess
N35.1 Postinfective urethral stricture, not elsewhere classified
N39.0 Urinary tract infection, site not specified
N41.0 Acute prostatitis
N41.2 Abscess of prostate
N43.1 Infected hydrocele
N45.0 Orchitis, epididymitis and epididymo-orchitis with abscess
N45.9 Orchitis, epididymitis and epididymo-orchits without abscess
N51.2 Balanitis in diseases classified elsewhere
N70.0 Acute salpingitis and oophoritis
N73.0 Acute parametritis and pelvic cellulitis
N73.1 Chronic parametritis and pelvic cellulitis
N73.2 Unspecified parametritis and pelvic cellulitis
N73.3 Female acute pelvic peritonitis
N73.4 Female chronic pelvic peritonitis
N73.9 Female pelvic inflammatory disease, unspecified
N74.2 Female syphilitic pelvic inflammatory disease
N74.3 Female gonococcal pelvic inflammatory disease
N74.4 Female chlamydial pelvic inflammatory disease
N75.0 Cyst of Bartholins gland
N75.1 Abscess of Bartholins gland
N76.0 Acute vaginitis
N76.4 Abscess of vulva
O03.0 Spontaneous abortion, incomplete, complicated by genital tract and pelvic infection
O03.5 Spontaneous abortion, complete or unspecified, complicated by genital tract and pelvic infection
O08.0 Genital tract and pelvic infection following ectopic and molar pregnancy
O26.4 Herpes gestationis
O86.2 Urinary tract infection following delivery
O98.1 Syphilis complicating pregnancy, childbirth and the puerperium
O98.2 Gonorrhea complicating pregnancy, childbirth and the puerperium
P39.3 Neonatal urinary tract infection
A80.1 Acute paralytic poliomyelitis, wild virus, imported
A80.2 Acute paralytic poliomyelitis, wild virus, indigenous
A80.3 Acute paralytic poliomyelitis, other and unspecified
A80.4 Acute nonparalytic poliomyelitis
A80.9 Acute poliomyelitis, unspecified
A81.8 Other atypical virus infections of central nervous system
A81.9 Atypical virus infection of central nervous system, unspecified
A82.0 Sylvatic rabies
A82.1 Urban rabies
A82.9 Rabies, unspecified
A83.0 Japanese encephalitis
A83.1 Western equine encephalitis
A83.2 Eastern equine encephalitis
A83.3 St Louis encephalitis
A83.4 Australian encephalitis
A83.5 California encephalitis

A83.6 Rocio virus disease
A83.8 Other mosquito-borne viral encephalitis
A83.9 Mosquito-borne viral encephalitis, unspecified
A84.0 Far Eastern tick-borne encephalitis [Russian spring-summer encephalitis]
A84.1 Central European tick-borne encephalitis
A84.8 Other tick-borne viral encephalitis
A84.9 Tick-borne viral encephalitis, unspecified
A85.0 Enteroviral encephalitis
A85.1 Adenoviral encephalitis
A85.2 Arthropod-borne viral encephalitis, unspecified
A85.8 Other specified viral encephalitis
A86 Unspecified viral encephalitis
A87.0 Enteroviral meningitis
A87.1 Adenoviral meningitis
A87.2 Lymphocytic choriomeningitis
A87.8 Other viral meningitis
A87.9 Viral meningitis, unspecified
A88.0 Enteroviral exanthematous fever
A88.8 Other specified viral infections of central nervous system
A89 Unspecified viral infection of central nervous system
A90 Dengue fever [classical dengue]
A91 Dengue haemorrhagic fever
A92.0 Chikungunya virus disease
A92.1 O'nyong-nyong fever
A92.2 Venezuelan equine fever
A92.3 West Nile virus infection
A92.4 Rift Valley fever
A92.8 Other specified mosquito-borne viral fevers
A92.9 Mosquito-borne viral fever, unspecified
A93.0 Oropouche virus disease
A93.2 Colorado tick fever
A93.8 Other specified arthropod-borne viral fevers
A94 Unspecified arthropod-borne viral fever
A95.0 Sylvatic yellow fever
A95.1 Urban yellow fever
A95.9 Yellow fever, unspecified
A96.0 Junin haemorrhagic fever
A96.1 Machupo haemorrhagic fever
A96.2 Lassa fever
A96.8 Other arenaviral hemorrhagic fevers
A96.9 Arenaviral haemorrhagic fever, unspecified
A98.0 Crimean-Congo hemorrhagic fever
A98.1 Omsk hemorrhagic fever
A98.2 Kyasanur Forest disease
A98.3 Marburg virus disease
A98.4 Ebola virus disease
A98.5 Hemorrhagic fever with renal syndrome
A98.8 Other specified viral haemorrhagic fevers
A99 Unspecified viral haemorrhagic fever
B00.1 Herpesviral vesicular dermatitis
B00.2 Herpesviral gingivostomatitis and pharyngotonsillitis
B00.3 Herpesviral meningitis
B00.4 Herpesviral encephalitis
B00.5 Herpesviral ocular disease
B00.7 Disseminated herpesviral disease
B00.8 Other forms of herpesviral infection
B00.9 Herpesviral infection, unspecified
B01.0 Varicella meningitis
B01.1 Varicella encephalitis
B01.8 Varicella with other complications
B01.9 Varicella without complication
B02.0 Zoster encephalitis
B02.1 Zoster meningitis
B02.2 Zoster with other nervous system involvement
B02.3 Zoster ocular disease
B02.7 Disseminated zoster
B02.8 Zoster with other complications
B02.9 Zoster without complications
B03 Smallpox
B04 Monkeypox
B05.0 Measles complicated by encephalitis
B05.1 Measles complicated by meningitis
B05.4 Measles with intestinal complications

B05.8 Measles with other complications
B05.9 Measles without complication
B06.0 Rubella with neurological complications
B06.8 Rubella with other complications
B06.9 Rubella without complication
B07 Viral warts
B08.0 Other orthopoxvirus infections
B08.1 Molluscum contagiosum
B08.2 Exanthema subitum [sixth disease]
B08.3 Erythema infectiosum [fifth disease]
B08.4 Enteroviral vesicular stomatitis with exanthem
B08.5 Enteroviral vesicular pharyngitis
B08.8 Other specified viral infections characterized by skin and mucous membrane lesions
B09 Unspecified viral infection characterized by skin and mucous membrane lesions
B15.0 Hepatitis A with hepatic coma
B15.9 Hepatitis A without hepatic coma
B16.0 Acute hepatitis B with delta-agent with hepatic coma
B16.1 Acute hepatitis B with delta-agent without hepatic coma
B16.2 Acute hepatitis B without delta-agent with hepatic coma
B16.9 Acute hepatitis B without delta-agent and without hepatic coma
B17.0 Acute delta-(super) infection of hepatitis B carrier
B17.1 Acute hepatitis C
B17.2 Acute hepatitis E
B17.8 Other specified acute viral hepatitis
B17.9 Acute viral hepatitis, unspecified
B18.0 Chronic viral hepatitis B with delta-agent
B18.1 Chronic viral hepatitis B without delta-agent
B18.2 Chronic viral hepatitis C
B18.8 Other chronic viral hepatitis
B18.9 Chronic viral hepatitis, unspecified
B19.0 Unspecified viral hepatitis with hepatic coma
B19.9 Unspecified viral hepatitis without hepatic coma
B20.0 HIV disease resulting in mycobacterial infection
B20.1 HIV disease resulting in other bacterial infections
B20.2 HIV disease resulting in cytomegaloviral disease
B20.3 HIV disease resulting in other viral infections
B20.4 HIV disease resulting in candidiasis
B20.5 HIV disease resulting in other mycoses
B20.6 HIV disease resulting in Pneumocystis jirovecii pneumonia
B20.7 HIV disease resulting in multiple infections
B20.8 HIV disease resulting in other infectious and parasitic diseases
B20.9 HIV disease resulting in unspecified infectious or parasitic
B21.0 HIV disease resulting in Kaposi sarcoma
B21.1 HIV disease resulting in Burkitt lymphoma
B21.2 HIV disease resulting in other types of non-Hodgkin lymphoma
B21.3 HIV disease resulting in other malignant neoplasms of lymphoid, haematopoietic and related tissue
B21.7 HIV disease resulting in multiple malignant neoplasms
B21.8 HIV disease resulting in other malignant neoplasms
B21.9 HIV disease resulting in unspecified malignant neoplasm
B22.0 HIV disease resulting in encephalopathy
B22.1 HIV disease resulting in lymphoid interstitial pneumonitis
B22.2 HIV disease resulting in wasting syndrome
B22.7 HIV disease resulting in multiple diseases classified elsewhere
B23.0 Acute HIV infection syndrome
B23.1 HIV disease resulting in (persistent) generalized lymphadenopathy
B23.2 HIV disease resulting in haematological and immunological abnormalities, not elsewhere classified
B23.8 HIV disease resulting in other specified conditions
B24 Unspecified human immunodeficiency virus [HIV] disease
B25.0 Cytomegaloviral pneumonitis
B25.1 Cytomegaloviral hepatitis
B25.2 Cytomegaloviral pancreatitis
B25.8 Other cytomegaloviral diseases
B25.9 Cytomegaloviral disease, unspecified
B26.0 Mumps orchitis
B26.1 Mumps meningitis
B26.2 Mumps encephalitis
B26.3 Mumps pancreatitis
B26.8 Mumps with other complications
B26.8 (no description found)
B26.9 Mumps without complication
B27.0 Gammaherpesviral mononucleosis
B27.1 Cytomegaloviral mononucleosis
B27.8 Other infectious mononucleosis

	B27.9 Infectious mononucleosis, unspecified<br>B30.0 Keratoconjunctivitis due to adenovirus<br>B30.1 Conjunctivitis due to adenovirus<br>B30.2 (no description found)<br>B30.3 Acute epidemic hemorrhagic conjunctivitis (enteroviral)<br>B30.8 Other viral conjunctivitis<br>B30.9 Viral conjunctivitis, unspecified<br>B33.0 Epidemic myalgia<br>B33.1 Ross River disease<br>B33.2 Viral carditis<br>B33.3 Retrovirus infections, not elsewhere classified<br>B33.4 Hantavirus (cardio-) pulmonary syndrome<br>B33.8 Other specified viral diseases<br>B34.1 Enterovirus infection, unspecified<br>B34.2 Coronavirus infection, unspecified site<br>B34.3 Parvovirus infection, unspecified site<br>B34.4 Papovavirus infection, unspecified<br>B34.8 Other viral infections of unspecified site<br>B34.9 Viral infection, unspecified<br>B97.0 Adenovirus as the cause of diseases classified to other chapters<br>B97.1 Enterovirus as the cause of diseases classified to other chapters<br>B97.2 Coronavirus as the cause of diseases classified to other chapters<br>B97.3 Retrovirus as the cause of diseases classified to other chapters<br>B97.4 Respiratory syncytial virus as the cause of diseases classified to other chapters<br>B97.5 Reovirus as the cause of diseases classified to other chapters<br>B97.6 Parvovirus as the cause of diseases classified to other chapters<br>B97.7 Papillomavirus as the cause of diseases classified to other chapters<br>B97.8 Other viral agents as the cause of diseases classified to other chapters<br>G02.0 Meningitis in viral diseases classified elsewhere<br>G05.1 Encephalitis, myelitis and encephalomyelitis in viral diseases classified elsewhere<br>H19.1 Herpesviral keratitis and keratoconjunctivitis<br>I41.1 Myocarditis in viral diseases classified elsewhere<br>J10.8 Influenza with other manifestations, influenza virus identified<br>J11 Influenza, virus not identified<br>J11.8 Influenza with other manifestations, virus not identified<br>M01.4 Rubella arthritis<br>M01.50 Arthritis in other viral diseases classified elsewhere, multiple sites<br>M01.51 Arthritis in other viral diseases classified elsewhere, shoulder region<br>M01.52 Arthritis in other viral diseases classified elsewhere, upper arm<br>M01.53 Arthritis in other viral diseases classified elsewhere, forearm<br>M01.54 Arthritis in other viral diseases classified elsewhere, hand<br>M01.55 Arthritis in other viral diseases classified elsewhere, pelvic region and thigh<br>M01.56 Arthritis in other viral diseases classified elsewhere, lower leg<br>M01.57 Arthritis in other viral diseases classified elsewhere, ankle and foot<br>M01.58 Arthritis in other viral diseases classified elsewhere, other site<br>M01.59 Arthritis in other viral diseases classified elsewhere, site unspecified<br>O98.4 Viral hepatitis complicating pregnancy, childbirth and the puerperium<br>O98.5 Other viral diseases complicating pregnancy, childbirth and the puerperium<br>P35.0 Congenital rubella syndrome<br>P35.1 Congenital cytomegalovirus infection<br>P35.2 Congenital herpesviral [herpes simples] infection<br>P35.3 Congenital viral hepatitis<br>P35.8 Other congenital viral diseases<br>P35.9 Congenital viral disease, unspecified<br>Z21 Asymptomatic human immunodeficiency virus [HIV] infection status
Respiratory non-infection	J30-J309 Vasomotor and allergic rhinitis<br>J31-J312 Chronic rhinitis, nasopharyngitis and pharyngitis<br>J32-J329 Chronic sinusitis<br>J33-J339 Nasal polyp<br>J34-J349 Other and unsepcified disorders of nose and nasal sinuses<br>J3501-J359 Chronic diseases of tonsils and adenoids<br>J36 Peritonsillar abscess<br>J37-J371 Chronic laryngitis and laryngotracheitis<br>J38-J387 Diseases of vocal cords and larynx, not elsewhere classified<br>J39-J399 Other diseases of upper respiratory tract<br>J40 Bronchitis, not specified as acute or chronic<br>J41 Simple and mucopurulent chronic bronchitis<br>J42 Unspecified chronic bronchitis<br>J43 Emphysema<br>J44 Other chronic obstructive pulmonary disease<br>J45 Asthma<br>J47 Bronchiectasis<br>J80 Acute respiratory distress syndrome

	J81 Pulmonary edema<br>J82 Pulmonary eosinophilia, not elsewhere classified<br>J84 Other interstitial pulmonary diseases<br>J85 Abscess of lung and mediastinum<br>J86 Pyothorax<br>J90 Pleural effusion, not elsewhere classified<br>J91 Pleural effusion in conditions classified elsewhere<br>J92 Pleural plaque<br>J93 Pneumothorax and air leak<br>J94 Other pleural conditions<br>J95 Intraoperative and postprocedural complications and disorders of respiratory system, not elsewhere classified<br>J96 Respiratory failure, not elsewhere classified<br>J98 Other respiratory disorders<br>J99 Respiratory disorders in diseases classified elsewhere
Gastrointestinal non-infection	K20 Esophagitis<br>K21 Gastro-esophageal reflux disease<br>K22 Other diseases of esophagus<br>K23 Disorders of esophagus in diseases classified elsewhere<br>K25 Gastric ulcer<br>K26 Duodenal ulcer<br>K27 Peptic ulcer, site unspecified<br>K28 Gastrojejunal ulcer<br>K29 Gastritis and duodenitis<br>K30 Functional dyspepsia<br>K31 Other diseases of stomach and duodenum<br>K90 Intestinal malabsorption<br>K91 Intraoperative and postprocedural complications and disorders of digestive system, not elsewhere classified<br>K92 Other diseases of digestive system<br>K40 Inguinal hernia<br>K41 Femoral hernia<br>K42 Umbilical hernia<br>K43 Ventral hernia<br>K44 Diaphragmatic hernia<br>K45 Other abdominal hernia<br>K46 Unspecified abdominal hernia<br>K50 Crohn's disease<br>K51 Ulcerative colitis<br>K52 Other and unspecified noninfective gastroenteritis and colitis<br>K55 Vascular disorders of intestine<br>K56 Paralytic ileus and intestinal obstruction without hernia<br>K57 Diverticular disease of intestine<br>K58 Irritable bowel syndrome<br>K59 Other functional intestinal disorders<br>K60 Fissure and fistula of anal and rectal regions<br>K61 Abcess of anal and rectal regions<br>K62 Other diseases of anus and rectum<br>K63 Other diseases of intestine<br>K65 Peritonitis<br>K66 Other disorders of peritoneum
Oral cavity	K00-K09 Diseases of oral cavity and salivary glands<br>K01 Dysphagia<br>K02-K029 Dental caries<br>K03-K039 Other diseases of hard tissues of teeth<br>K04-K0499 Diseases of pulp and periapical tissues<br>K05-K056 Gingivitis and periodontal diseases<br>K06-K069 Other disorders of gingiva and eentulous alveolar ridge<br>K08-K089 Other disorders of teeth and supporting structures<br>K09-K099 Cysts of oral region, not elsewhere classified<br>K11-K119 Diseases of salivary glands<br>K12-K1239 Stomatitis and related lesions<br>K13-K1379 Other diseases of lip and oral mucosa<br>K14-K149 Diseases of tongue<br>R1310 Dysphagia<br>Z012 Encounter for dental examination and cleaning<br>Z463 Encounter for fitting and adjustment of dental prosthetic device<br>Z464 Encounter for fitting and adjustment of orthodontic device
Genitourinary non-infection	N00 Acute nephritic syndrome<br>N01 Rapidly progressive nephritic syndrome<br>N02 Recurrent and persistent haematuria<br>N03 Chronic nephritic syndrome<br>N04 Nephrotic syndrome<br>N05 Unspecified nephritic syndrome<br>N06 Isolated proteinuria with specified morphological lesion

	N07 Hereditary nephropathy, not elsewhere classified
	N08 Glomerular disorders in diseases classified elsewhere
	N10 Acute tubulo-interstitial nephritis
	N11 Chronic tubulo-interstitial nephritis
	N12 Tubulo-interstitial nephritis, not specified as acute or chronic
	N13 Obstructive and reflux uropathy
	N14 Drug- and heavy-metal-induced tubulo-interstitial and tubular conditions
	N15 Other renal tubulo-interstitial diseases
	N16 Renal tubulo-interstitial disorders in diseases classified elsewhere
	N17 Acute renal failure
	N18 Chronic renal failure
	N19 Unspecified renal failure
	N20 Calculus of kidney and ureter
	N21 Calculus of lower urinary tract
	N22 Calculus of urinary tract in diseases classified elsewhere
	N23 Unspecified renal colic
	N25 Disorders resulting from impaired renal tubular function
	N26 Unspecified contracted kidney
	N27 Small kidney of unknown cause
	N28 Other disorders of kidney and ureter, not elsewhere classified
	N29 Other disorders of kidney and ureter in diseases classified elsewhere
	N30 Cystitis
	N31 Neuromuscular dysfunction of bladder, not elsewhere classified
	N32 Other disorders of bladder
	N33 Bladder disorders in diseases classified elsewhere
	N34 Urethritis and urethral syndrome
	N35 Urethral stricture
	N36 Other disorders of urethra
	N37 Urethral disorders in diseases classified elsewhere
	N39 Other disorders of urinary system
	N40 Hyperplasia of prostate
	N41 Inflammatory diseases of prostate
	N42 Other disorders of prostate
	N43 Hydrocele and spermatocele
	N44 Torsion of testis
	N45 Orchitis and epididymitis
	N46 Male infertility
	N47 Redundant prepuce, phimosis and paraphimosis
	N48 Other disorders of penis
	N49 Inflammatory disorders of male genital organs, not elsewhere classified
	N50 Other disorders of male genital organs
	N51 Disorders of male genital organs in diseases classified elsewhere
	N60 Benign mammary dysplasia
	N61 Inflammatory disorders of breast
	N62 Hypertrophy of breast
	N63 Unspecified lump in breast
	N64 Other disorders of breast
	N70 Salpingitis and oophoritis
	N71 Inflammatory disease of uterus, except cervix
	N72 Inflammatory disease of cervix uteri
	N73 Other female pelvic inflammatory diseases
	N74 Female pelvic inflammatory disorders in diseases classified elsewhere
	N75 Diseases of Bartholin's gland
	N76 Other inflammation of vagina and vulva
	N77 Vulvovaginal ulceration and inflammation in diseases classified elsewhere
	N80 Endometriosis
	N81 Female genital prolapse
	N82 Fistulae involving female genital tract
	N83 Noninflammatory disorders of ovary, fallopian tube and broad ligament
	N84 Polyp of female genital tract
	N85 Other noninflammatory disorders of uterus, except cervix
	N86 Erosion and ectropion of cervix uteri
	N87 Dysplasia of cervix uteri
	N88 Other noninflammatory disorders of cervix uteri
	N89 Other noninflammatory disorders of vagina
	N90 Other noninflammatory disorders of vulva and perineum
	N91 Absent, scanty and rare menstruation
	N92 Excessive, frequent and irregular menstruation
	N93 Other abnormal uterine and vaginal bleeding
	N94 Pain and other conditions associated with female genital organs and menstrual cycle
	F90-F909 Attention-deficit hyperactivity disorders
Mental health	F91-F919 Conduct disorders
	F93-F939 Emotional disorders with onset specific to childhood

	F94-F949 Disorders of social functioning with onset specific to childhood and adolescence<br>F95-F959 Tic disorder<br>F98-F989 Other behavioral and emotional disorders with onset usually occurring in childhood and adolescence<br>F99 Mental disorder, not otherwise specified<br>F40-F409 Phobic anxiety disorders<br>F41-F419 Other anxiety disorders<br>F422-F429 Obsessive-compulsive disorder<br>F43-F439 Reaction to severe stress, and adjustment disorders<br>F481-F489 Other nonpsychotic mental disorders
Injury/External	J60 Coalworker's pneumoconiosis<br>J61 Pneumoconiosis due to asbestos and other mineral fibres<br>J62 Pneumoconiosis due to dust containing silica<br>J63 Pneumoconiosis due to other inorganic dusts<br>J64 Unspecified pneumoconiosis<br>J65 Pneumoconiosis associated with tuberculosis<br>J66 Airway disease due to specific organic dust<br>J67 Hypersensitivity pneumonitis due to organic dust<br>J68 Respiratory conditions due to inhalation of chemicals, gases, fumes and vapours<br>J69 Pneumonitis due to solids and liquids<br>J70 Respiratory conditions due to other external agents<br>S00-S09 Injuries to the head<br>S10-S19 Injuries to the neck<br>S20-S29 Injuries to the thorax<br>S30-S39 Injuries to the abdomen, lower back, lumbar spine and pelvis<br>S40-S49 Injuries to the shoulder and upper arm<br>S50-S59 Injuries to the elbow and forearm<br>S60-S69 Injuries to the wrist and hand<br>S70-S79 Injuries to the hip and thigh<br>S80-S89 Injuries to the knee and lower leg<br>S90-S99 Injuries to the ankle and foot<br>T00-T07 Injuries involving multiple body regions<br>T08-T14 Injuries to unspecified parts of trunk, limb or body region<br>T15-T19 Effects of foreign body entering through natural orifice<br>T20-T32 Burns and corrosions<br>T33-T35 Frostbite<br>T36-T50 Poisoning by drugs, medicaments and biological substances<br>T51-T65 Toxic effects of substances chiefly nonmedicinal as to source<br>T66-T78 Other and unspecified effects of external causes<br>T79 Certain early complications of trauma<br>T80-T88 Complications of surgical and medical care, not elsewhere classified<br>T90-T98 Sequelae of injuries, of poisoning and of other consequences of external causes<br>P10-P15 Birth trauma<br>V01-V99 Transport accidents<br>W00-X59 Other external causes of accidental injury<br>Y85-Y89 Sequelae of external causes of morbidity and mortality
Congenital anomalies	Q00-Q07 Congenital malformations of the nervous system<br>Q10-Q18 Congenital malformations of eye, ear, face and neck<br>Q20-Q28 Congenital malformations of the circulatory system<br>Q30-Q34 Congenital malformations of the respiratory system<br>Q35-Q37 Cleft lip and cleft palate<br>Q38-Q45 Other congenital malformations of the digestive system<br>Q50-Q56 Congenital malformations of genital organs<br>Q60-Q64 Congenital malformations of the urinary system<br>Q65-Q79 Congenital malformations and deformations of the musculoskeletal system<br>Q80-Q89 Other congenital malformations<br>Q90-Q99 Chromosomal abnormalities, not elsewhere classified
Neoplasm	C00-C96Z Malignant neoplasm<br>D00-D099 In situ neoplasm<br>D10-D369 Benign neoplasm<br>D3701-D489 Neoplasm of uncertain behaviour<br>D3A00-D3A8 Benign tumours<br>D49-D499 Neoplasms of uncertain or unknown behaviour

ICD-10 International Classification of Diseases10th revision.

Supplementary Table 9 | Strengthening the Reporting of Observational Studies in Epidemiology (STROBE) reporting guideline

	Item no.	Recommendation	Page no.	Relevant text from manuscript
**Title and abstract**	1	(*a*) Indicate the study's design with a commonly used term in the title or the abstract	P1-5	Summary
		(*b*) Provide in the abstract an informative and balanced summary of what was done and what was found	P1-5	Summary
**Introduction**				
Background/Rationale	2	Explain the scientific background and rationale for the investigation being reported	P6-7	Introduction
Objectives	3	State specific objectives, including any prespecified hypotheses	P6-7	Introduction
**Methods**				
Study design	4	Present key elements of study design early in the paper	P17-21	Method
Setting	5	Describe the setting, locations, and relevant dates, including periods of recruitment, exposure, follow-up, and data collection	P17-21	Method
Participants	6	(a) *Cohort study*—Give the eligibility criteria, and the sources and methods of selection of participants. Describe methods of follow-up<br>*Case-control study*—Give the eligibility criteria, and the sources and methods of case ascertainment and control selection. Give the rationale for the choice of cases and controls<br>*Cross-sectional study*—Give the eligibility criteria, and the sources and methods of selection of participants		
		(*b*) *Cohort study*—For matched studies, give matching criteria and number of exposed and unexposed<br>*Case-control study*—For matched studies, give matching criteria and the number of controls per case	P17-21	Method
Variables	7	Clearly define all outcomes, exposures, predictors, potential confounders, and effect modifiers. Give diagnostic criteria, if applicable	P17-21	Method
Data sources/Measurement	8[a]	For each variable of interest, give sources of data and details of methods of assessment (measurement). Describe comparability of assessment methods if there is more than one group	P17-21	Method
Bias	9	Describe any efforts to address potential sources of bias	P17-21	Method
Study size	10	Explain how the study size was arrived at	P17-21	Method
Quantitative variables	11	Explain how quantitative variables were handled in the analyses. If applicable, describe which groupings were chosen and why	P17-21	Method
Statistical methods	12	(*a*) Describe all statistical methods, including those used to control for confounding	P17-21	Method
		(*b*) Describe any methods used to examine subgroups and interactions	P17-21	Method
		(*c*) Explain how missing data were addressed	P17-21	Method
		(*d*) *Cohort study*—If applicable, explain how loss to follow-up was<br>*Case-control study*—If applicable, explain how matching of cases and controls was addressed<br>*Cross-sectional study*—If applicable, describe analytical methods taking account of sampling strategy addressed	P17-21	Method
		(*e*) Describe any sensitivity analyses	P17-21	Method
**Results**				
Participants	13[a]	(a) Report numbers of individuals at each stage of study—e.g., numbers potentially eligible, examined for eligibility, confirmed eligible, included in the study, completing follow-up, and analysed	P8-9	Results
		(b) Give reasons for non-participation at each stage	P8-9	Results
		(c) Consider use of a flow diagram	P8-9	Results

Descriptive data	14[a]	(a) Give characteristics of study participants (e.g., demographic, clinical, social) and information on exposures and potential confounders	P8-9	Results
		(b) Indicate number of participants with missing data for each variable of interest	P8-9	Results
		(c) *Cohort study*—Summarise follow-up time (e.g., average and total amount)	P8-9	Results
Outcome data	15[a]	*Cohort study*—Report numbers of outcome events or summary measures over time	P8-9	Results
		Case-control study—Report numbers in each exposure category, or summary measures of exposure		
		Cross-sectional study—Report numbers of outcome events or summary measures		
Main results	16	(*a*) Give unadjusted estimates and, if applicable, confounder-adjusted estimates and their precision (e.g., 95% confidence interval). Make clear which confounders were adjusted for and why they were included	P8-9	Results
		(*b*) Report category boundaries when continuous variables were categorized	P8-9	Results
		(*c*) If relevant, consider translating estimates of relative risk into absolute risk for a meaningful time period	P8-9	Results
Other analyses	17	Report other analyses done—e.g., analyses of subgroups and interactions, and sensitivity analyses		
**Discussion**				
Key results	18	Summarise key results with reference to study objectives		
Limitations	19	Discuss limitations of the study, taking into account sources of potential bias or imprecision. Discuss both direction and magnitude of any potential bias	P10-16	Discussion
Interpretation	20	Give a cautious overall interpretation of results considering objectives, limitations, multiplicity of analyses, results from similar studies, and other relevant evidence	P10-16	Discussion
Generalisability	21	Discuss the generalisability (external validity) of the study results	P10-16	Discussion
**Other information**				
Funding	22	Give the source of funding and the role of the funders for the present study and, if applicable, for the original study on which the present article is based	P22	Funding

An Explanation and Elaboration article discusses each checklist item and gives methodological background and published examples of transparent reporting. The STROBE checklist is best used in conjunction with this article (freely available on the web sites of PLoS Medicine at http://www.plosmedicine.org/, Annals of Internal Medicine at http://www.annals.org/, and Epidemiology at http://www.epidem.com/). Information on the STROBE initiative is available at www.strobe-statement.org.

[a]Give information separately for cases and controls in case-control studies and, if applicable, for exposed and unexposed groups in cohort and cross-sectional studies.
