# Supplementary material for: Breastfeeding and impact on childhood hospital admissions: a nationwide birth cohort in South Korea
Source: Nat Commun. 2023 Sep 20;14:5819. doi: 10.1038/s41467-023-41516-y (PMC10511528; doi:10.1038/s41467-023-41516-y)
Supplement: Supplementary file 2 — Reporting Summary [file 41467_2023_41516_MOESM2_ESM.pdf]

nature portfolio

Corresponding author(s): Dong Keon Yon
Last updated by author(s): Aug 15, 2023

Reporting Summary

Nature Portfolio wishes to improve the reproducibility of the work that we publish. This form provides structure for consistency and transparency in reporting. For further information on Nature Portfolio policies, see our Editorial Policies and the Editorial Policy Checklist.

Statistics

For all statistical analyses, confirm that the following items are present in the figure legend, table legend, main text, or Methods section.

n/a	Confirmed	
☐	☒	The exact sample size (*n*) for each experimental group/condition, given as a discrete number and unit of measurement
☐	☒	A statement on whether measurements were taken from distinct samples or whether the same sample was measured repeatedly
☐	☒	The statistical test(s) used AND whether they are one- or two-sided<br>*Only common tests should be described solely by name; describe more complex techniques in the Methods section.*
☐	☒	A description of all covariates tested
☐	☒	A description of any assumptions or corrections, such as tests of normality and adjustment for multiple comparisons
☐	☒	A full description of the statistical parameters including central tendency (e.g. means) or other basic estimates (e.g. regression coefficient) AND variation (e.g. standard deviation) or associated estimates of uncertainty (e.g. confidence intervals)
☐	☒	For null hypothesis testing, the test statistic (e.g. *F*, *t*, *r*) with confidence intervals, effect sizes, degrees of freedom and *P* value noted<br>*Give P values as exact values whenever suitable.*
☒	☐	For Bayesian analysis, information on the choice of priors and Markov chain Monte Carlo settings
☒	☐	For hierarchical and complex designs, identification of the appropriate level for tests and full reporting of outcomes
☒	☐	Estimates of effect sizes (e.g. Cohen's *d*, Pearson's *r*), indicating how they were calculated

Our web collection on statistics for biologists contains articles on many of the points above.

Software and code

Policy information about availability of computer code

Data collection	Data collection were performed using SAS (version 9.4; SAS Institute Inc., Cary, NC, USA) for big-data analysis.
Data analysis	Statistical analyses were performed using R software (version 3.1.1; R Foundation, Vienna, Austria) for generating figures, Statistical Package for Social Sciences (version 25.0; IBM Corp, Armonk, NY, USA) for exploratory data analysis, and SAS (version 9.4; SAS Institute Inc., Cary, NC, USA) for big-data analysis. A nearest-neighbor algorithm was used to match infants in two groups with a random selection without replacement within specified caliper widths (0.001 standard deviations), using SAS.

For manuscripts utilizing custom algorithms or software that are central to the research but not yet described in published literature, software must be made available to editors and reviewers. We strongly encourage code deposition in a community repository (e.g. GitHub). See the Nature Portfolio guidelines for submitting code & software for further information.

Data

Policy information about availability of data

All manuscripts must include a data availability statement. This statement should provide the following information, where applicable:

- Accession codes, unique identifiers, or web links for publicly available datasets
- A description of any restrictions on data availability
- For clinical datasets or third party data, please ensure that the statement adheres to our policy

The datasets analysed during the current study are available in the National Health Insurance Service, South Korea, https://nhiss.nhis.or.kr/bd/ab/bdaba000eng.do. This protects the confidentiality of the data and ensures that Information Governance is robust. Applications to access health data in South Korea are submitted to

the National Health Insurance Service, South Korea. Information can be found at https://nhiss.nhis.or.kr/bd/ab/bdaba000eng.do. Source data (Figure 3) are provided with this paper.

Human research participants

Policy information about studies involving human research participants and Sex and Gender in Research.

Reporting on sex and gender	This study was based on the National Health Insurance Service in the Republic of Korea. We selected the dataset of all infants born between 1 January 2010 and 31 December 2017, and who had records of receiving the first National Health Screening Program for Infants and Children. Thus, this is determined by the "sex" of a born child reported to the government system.
Population characteristics	The dataset was linked and consisted of data on first general health examination results, death records, health insurance data including insurance eligibility data, personal sociodemographic data, inpatient and outpatient healthcare records, and medication records. Models were adjusted for infant sex, calendar period of birth (2009-2010, 2011-2012, and 2013-2015), birth season (spring, summer, autumn, or winter), region of residence (rural or urban)32,33,41, household income (high, middle, or low), preterm birth (≤36 weeks), and low birth weight (≤2499 g). When considering the scope of this study, it has been taken into account that the research targets infants. Therefore, instead of using the age of the infants, the calendar period of birth has been considered as a proxy for age.
Recruitment	The study was a population-based, nationwide birth cohort study consisting of all Korean infants born between 1 January 2009 and 31 December 2015, and who received the first National Health Screening Program for Infants and Children (n = 2,010,325). The Korean government provides a complimentary first general health examination to all Korean infants aged 6 months. Among 2,010,325 infants in South Korea, we excluded infants (n = 401,785) diagnosed with a congenital anomaly in the first 6 months of life and those diagnosed with malignant neoplasm, a blood disorder, chronic kidney disease, cystic fibrosis, and/or immune dysfunction. The final sample size was 1,608,540 infants. In order to minimize selection bias, we targeted all infants born in South Korea between 2009 and 2015 as our study population.
Ethics oversight	The study was approved by the Institutional Review Board of Sejong University (Seoul, South Korea; SJU-HR-E-2021-001) and Seoul National University (Seoul, South Korea; E-2108-134-1246). The Korean National Health Insurance Service and the Korean Government provided information governance approval (NHIS-2022-1-383). Under the terms of the approval, patient consent was not required for use of routine health records for our study.

Note that full information on the approval of the study protocol must also be provided in the manuscript.

Field-specific reporting

Please select the one below that is the best fit for your research. If you are not sure, read the appropriate sections before making your selection.

[x] Life sciences [ ] Behavioural & social sciences [ ] Ecological, evolutionary & environmental sciences

For a reference copy of the document with all sections, see nature.com/documents/nr-reporting-summary-flat.pdf

Life sciences study design

All studies must disclose on these points even when the disclosure is negative.

Sample size	The study was a population-based, nationwide birth cohort study consisting of all Korean infants born between 1 January 2009 and 31 December 2015, and who received the first National Health Screening Program for Infants and Children (n = 2,010,325). The Korean government provides a complimentary first general health examination to all Korean infants aged 6 months. In order to investigate the association between breastfeeding and hospitalization rates, we aimed to minimize selection bias by including the entire national population as our study sample.
Data exclusions	Among 2,010,325 infants in South Korea, we excluded infants (n = 401,785) diagnosed with a congenital anomaly in the first 6 months of life and those diagnosed with malignant neoplasm, a blood disorder, chronic kidney disease, cystic fibrosis, and/or immune dysfunction. The final sample size was 1,608,540 infants.
Replication	We performed the negative binomial regression model (endpoint, incidence rate of any hospital admission) with IRRs and 95% confidence intervals (CIs). Models were adjusted for infant sex, calendar period of birth (2009-2010, 2011-2012, and 2013-2015), birth season (spring, summer, autumn, or winter), region of residence (rural or urban), household income (high, middle, or low), preterm birth (≤36 weeks), and low birth weight (≤2499 g). In order to ensure the reproducibility of the study, various subgroup and sensitivity analyses were performed: a stratification analysis based on hospital admission risk at different time points (less than 1 year, 1 to 2 years, 3 to 4 years, 5 to 6 years, and 7 to 10 years) or on cause of morbidity (infection, non-infection respiratory, non-infection gastrointestinal tract, non-infection genitourinary tract, non-infection oral cavity, mental health, and injury/external); a subgroup analysis according to infant sex, calendar period of birth, and region of residence; and alternative cohort specifications (propensity-score-matched cohort). To investigate potential heterogeneity in the effects of breastfeeding on hospitalization outcomes, we have decided to conduct three additional subgroup analyses (age, calendar period of birth, and region of residence) based on existing literature or biological hypothesis. In additional, we used a propensity score-matched cohort to determine robustness and generalization of our main results. We performed 1:1 exposure-driven propensity score matching to balance the baseline covariates of two groups to minimize potential confounding effects. Propensity scores were derived using a multivariable logistic regression model from the predicted probability of exclusively breastfed infants vs. fully formula-fed infants (each n = 600,988).

Randomization	Not applicable; individuals were recruited in an observational study with convenience samples being collected.
Blinding	Blinding was not relevant, since this is an observational study, where participants were invited based on test status (case or control)

Reporting for specific materials, systems and methods

We require information from authors about some types of materials, experimental systems and methods used in many studies. Here, indicate whether each material, system or method listed is relevant to your study. If you are not sure if a list item applies to your research, read the appropriate section before selecting a response.

Materials & experimental systems

n/a	Involved in the study
☒	☐ Antibodies
☒	☐ Eukaryotic cell lines
☒	☐ Palaeontology and archaeology
☒	☐ Animals and other organisms
☒	☐ Clinical data
☒	☐ Dual use research of concern

Methods

n/a	Involved in the study
☒	☐ ChIP-seq
☒	☐ Flow cytometry
☒	☐ MRI-based neuroimaging
